# Food Insecurity and Maternal Diet Influence Human Milk Composition between the Infant’s Birth and 6 Months after Birth in Central-Africa

**DOI:** 10.3390/nu14194015

**Published:** 2022-09-27

**Authors:** Jeanne H. Bottin, Simone R. B. M. Eussen, Aisosa J. Igbinijesu, Marko Mank, Jean-Christophe Junior Koyembi, Yawo Tufa Nyasenu, Gilles Ngaya, Daniel Mad-Bondo, Jean-Bertrand Kongoma, Bernd Stahl, Philippe J. Sansonetti, Raphaëlle Bourdet-Sicard, Violeta Moya-Alvarez

**Affiliations:** 1 Danone Nutricia Research, 91120 Palaiseau, France; 2 Danone Nutricia Research, 3584 CT Utrecht, The Netherlands or; 3 Unité d’Epidémiologie, Institut Pasteur de Bangui, Bangui BP923, Central African Republic; 4 Laboratoire d’Analyses Médicales, Institut Pasteur de Bangui, Bangui BP923, Central African Republic; 5 Laboratoire de Biologie Moléculaire et d’Immunologie, Université de Lomé, Lomé P.O. Box 1396, Togo; 6 Direction du Service de Santé de la Gendarmerie, Sis Camp Henri IZAMO, Bangui BP790, Central African Republic; 7 Department of Chemical Biology & Drug Discovery, Utrecht Institute for Pharmaceutical Sciences, Utrecht University, 3584 CG Utrecht, The Netherlands; 8 Chaire de Microbiologie et Maladies Infectieuses, Collège de France, 75005 Paris, France; 9 Unité de Pathogénie Microbienne Moléculaire, INSERM U1202, Department of Cell Biology and Infection, Institut Pasteur, 75015 Paris, France; 10 Epidemiology of Emergent Diseases Unit, Global Health Department, Institut Pasteur, 75015 Paris, France

**Keywords:** human milk, human milk oligosaccharides (HMOs), Africa, fatty acids, amino acids

## Abstract

Although the World Health Organization (WHO) and UNICEF recommend that infants should be exclusively breastfed for the first 6 months of life, evidence is scarce on how the mother’s undernourishment status at delivery and maternal dietary factors influence human milk (HM) composition during the first 6 months of life in regions with high food insecurity. The maternal undernourishment status at delivery, maternal diet, and HM nutrients were assessed among 46 women and their 48 vaginally born infants in Bangui at 1, 4, 11, 18, and 25 weeks after birth through 24-h recalls and food consumption questionnaires from December 2017 to June 2019 in the context of the "Mother-to-Infant TransmIssion of microbiota in Central-Africa" (MITICA) study. High food insecurity indexes during the follow-up were significantly associated with them having lower levels of many of the human milk oligosaccharides (HMOs) that were measured and with lower levels of retinol (aß-coef = −0.2, *p* value = 0.04), fatty acids (aß-coef = −7.2, *p* value = 0.03), and amino acids (aß-coef = −2121.0, *p* value < 0.001). On the contrary, women from food-insecure households displayed significantly higher levels of lactose in their HM (aß-coef = 3.3, *p* value = 0.02). In parallel, the consumption of meat, poultry, and fish was associated with higher HM levels of many of the HMOs that were measured, total amino acids (aß-coef = 5484.4, *p* value < 0.001), and with lower HM levels of lactose (aß-coef = −15.6, *p* value = 0.01). Food insecurity and maternal diet had a meaningful effect on HM composition with a possible impact being an infant undernourishment risk. Our results plead for consistent actions on food security as an effective manner to influence the nutritional content of HM and thereby, potentially improve infant survival and healthy growth.

## 1. Introduction

The World Health Organization (WHO) and UNICEF recommend that children initiate breastfeeding within the first hour after birth and be exclusively breastfed for the first six months of life [1]. This is of special concern in developing countries where infants face higher environmental and nutritional challenges due to their limited access to clean water and sanitation, and the high infectious and nutritional disease burden. Indeed, the optimal breastfeeding of infants from birth to 24 months has the potential to prevent over 800,000 deaths (13 percent of all deaths) in children under five years, worldwide [2]. 

As the primary nutritional source for neonates, the human milk’s (HM) composition is of paramount importance for the neonate’s survival and development. In addition to its immunologically active molecules and bioactive compounds that provide immediate protection against life-threatening conditions such as necrotizing enterocolitis [1,3], HM provides a myriad of nutrients that are essential for optimal development in early life [4,5]. Precisely, HM fulfills the energy and nutrient requirements that an infant has during the first six months of life. After this, it continues to provide up to half or more of a child’s necessary nutrients during the rest of the first year, and up to one third during the second year of life [1]. Due to the tremendous beneficial effects of breastfeeding for infant survival and growth globally, the raising interest in optimizing infant nutrition during early life has brought a plethora of studies that have investigated the main determinants of HM composition.

Research from Germany [6,7], Italy [8,9], China [10], New Zealand [11,12], Iceland [13], India [14], and Spain [15] has revealed that HM composition varies depending on maternal food consumption habits, in addition to the maternal gestational age, lactation period, genetics, and life-style [16,17]. Moreover, maternal diet may play a role in shaping the infant gut microbial colonization by modulating the HM composition [15,18]. Albeit this research evidence, the effect of the maternal diet and the maternal undernourishment status at delivery on the HM nutritional composition has not yet been analyzed in a context of high food insecurity. 

The Central-African Republic lacks precise undernourishment estimates among pregnant women, even though anemia estimates were over 51.5% in 2019 [19]. However, the Central-African region is plagued by food insecurity [20]. For these reasons, we selected the Bangui region to conduct this prospective, observational study. Precisely, the “Mother-to-Infant TransmIssion of microbiota in Central-Africa” (MITICA) study addressed the effect of maternal undernourishment on infant malnutrition, with HM and microbes as a possible link between them. In this article, we aimed to decipher how maternal dietary factors and maternal undernourishment status at delivery might influence the HM composition from the infant’s birth until 6 months, i.e., the timeframe that corresponds to the WHO recommendations on exclusive breastfeeding. Evaluating the differences in the HM nutrients (carbohydrates, fatty acids (FAs), amino acids (AAs), human milk oligosaccharides (HMOs), and retinol) depending on their household food security status, maternal dietary factors, and maternal nutritional status at delivery may help us to better understand the impact of maternal undernutrition and food insecurity on HM composition and infant health during the postnatal period. 

## 2. Materials and Methods

### 2.1. Study Design

Forty-eight women and their 50 vaginally born infants were followed in Bangui from 8 December 2017 to 29 June 2019 in the context of the “Mother-to-Infant TransmIssion of microbiota in Central-Africa” (MITICA) study. Over 200 pregnant women were pre-included either at antenatal care (ANC) visits or in the neighborhoods surrounding the Henri-Izamo maternity facility in Bangui. The recruitment period (December 2017–June 2019) was set in advance due to logistic and financial constraints. All of the women delivering within the laboratory opening hours (8 AM–2 PM) with negative rapid diagnostic test results for HIV, HBV, and HCV at delivery were de facto included in the cohort. Right after delivery, an extended questionnaire on their pregnancy history, the socio-economic status of the household, woman empowerment status, food consumption, and food security status was filled in, in Sango, which is the local language. Additionally, anthropometric measures of the mother and infant were taken at the point of delivery. Furthermore, 8 mL venous blood and 4 mL cord blood were drawn from the mother and the newborn, respectively. The cord blood sample was drawn immediately after cutting the cord from the newborn’s side. After delivery, systematic visits were scheduled at 1, 4, 11, 18, and 25 weeks after birth. At all of the systematic visits, 8 mL HM were collected, in parallel to a 24-h food recall, a food consumption questionnaire, and a questionnaire on the hygienic measures of the household. Due to the close visits at the beginning of the follow-up (birth and one week after birth), the food consumption questionnaires were only addressed once after the delivery (starting at 1 week after delivery).

The MITICA study fulfilled the good practices of the Declaration of Helsinki and was approved by the Institutional Review Board (IRB) of the Pasteur Institute (2016-09/IRB) on 28 April 2017, the Ethics Committee of the Faculty of Sciences of Bangui (9/UB/FACSS/CSVPR/17) on 10 April 2017, and the Ministry of Health of the Central-African Republic (189/MSP/DIRCAB/DGPGHU/DGEHU) on 9 June 2017. Informed consent was gathered at the pre-inclusion after a detailed explanation by the clinical research associate, and it was then confirmed at the delivery. More precise details on the MITICA study can be found elsewhere [21].

### 2.2. Assessment of Maternal Diet

Maternal diet composition and feeding practices were assessed by analyzing: (i) a 24-h recall questionnaire; (ii) a food consumption questionnaire (including feeding practices); (iii) a food security questionnaire at each follow-up visit. The questionnaires are presented in the supplementary data (Table S1). Briefly, the 24-h recall is an interview that gathers all information about all of the food and portions that were consumed the previous day. The food consumption questionnaire collects information on the different food categories that were eaten by the mother the previous day in a closed, standard questionnaire format following the FAO recommendations [22]. This questionnaire considers meat, poultry, and fish as a joint category. As the literature reports that fatty acid levels are associated with fish consumption, fish was also analyzed separately in these analyses to improve the accuracy of the statistical associations. The Women’s Dietary Diversity Score (WDDS) was obtained for each woman from both the 24-h recall questionnaire and the food consumption questionnaire following the same FAO guidelines [22]. Additional information on hygienic measures was also gathered. The Household Food Insecurity Access Scale (HFIAS) and Household Hunger Scale (HHS) were calculated for measuring the food security of the household [23,24]. The HFIAS is composed of a set of nine questions that appear to distinguish food-insecure from food-secure households across different cultural settings. The HFIAS is used to assess the access component of the prevalence of household food insecurity and to detect changes in food insecurity over time. The HFIAS categories correspond to no food insecurity, mild food insecurity, moderate food insecurity, and severe food insecurity. The HHS has been specifically validated to measure household hunger in food-insecure areas. Moreover, it produces valid and comparable results across different cultures and settings so that the status of different population groups can be described in a meaningful and comparable way. It is divided into little to no hunger in the household, moderate hunger in the household, and severe hunger in the household.

The undernourishment status of the women at the point of delivery was determined using their albumin plasma levels (<35 g/L) according to the international standard cut-off values [25,26].

### 2.3. Human Milk Sampling and Analyses

Between 10 AM and noon, and at least two hours after the previous breast feed, 8 mL of foremilk HM were poured manually by the mother into a sterile tube before breastfeeding the infant at the Institut Pasteur de Bangui (IPB). The foremilk HM samples were immediately transferred into a −80 °C freezer, and then sent to the Danone Nutricia Research laboratory facilities in Utrecht, the Netherlands on dry-ice via the Institut Pasteur in Paris, where the HM was pasteurized to avoid any possible infectious contamination.

At the Danone Nutricia Research facility, the HM samples were thawed overnight at 4 °C, whereupon they were gently vortexed and aliquoted. Two 250 µL HM aliquots were analyzed for either amino acid (AA) or fatty acid (FA) concentrations by the standard methods that are described in detail elsewhere [27,28,29]. The HM samples were spiked prior to a lipid extraction [30] with C19:0 to enable FA quantification. The FA concentration was analyzed using a gas chromatograph (GC) that was equipped with a flame ionization detector (FID); the processing and derivatization processes were conducted according to Morrison and Smith [27]. For the determination of poly-unsaturated fatty acids (PUFA), a known amount of C19:0 PC was added as an internal standard to 100 µL sample (HM). The lipids were converted to fatty acid methyl esters with methanol + 2% sulphuric acid at 100 °C for 60 minutes. The fatty acid methyl esters (FAMEs) were extracted with hexane and, after an evaporation procedure was performed, they were dissolved in isooctane. One µL of the isooctane was injected into the GC. The FAMEs were separated on a CP-Sil 88 column and detected using a FID detector. The FAME identification was based on their retention time. The relative concentration of them was based on the peak area, and the absolute concentration of them was calculated after their normalization with the C19:0 peak.

Free amino acids (FAA) and total amino acids (TAA) were also determined. The TAA included protein-bound ones and FAA. The determination of the TAA required a prior protein hydrolysis, and it covered 15 detectable AA or AA groups. The acidic hydrolysis process converts asparagine into aspartate (when they are combined, this is referred to as Asx) and glutamine into glutamate (when they are combined, this is referred to as Glx). The FAA analysis does not allow the detection of tryptophan (Trp), cysteine (Cys), and proline (Pro), thereby yielding a total of 18 detectable FAAs and taurine. The methods that were used were based on Teerlink et al. [29]. Precisely, the TAAs and FAAs were analyzed as follows: The proteins in the sample were completely broken down to amino acids by an acid hydrolysis procedure with hydrochloric acid. The amount of the separate amino acids in the hydrolysate was determined by a UFLC procedure using a pre-column derivatization with o-phtaldialdehyde and a fluorimetry procedure. For the FAA determination, proteins and polypeptides were precipitated with perchloric acid, and the sample was then centrifuged. The content of the individual amino acids was determined by UFLC using a pre-column derivatization with o-phtaldialdehyde and a fluorimetry procedure for their detection.

To analyse the retinol levels, the HM aliquots were treated at ambient temperature with an ethanolic potassium hydroxide solution for 15–20 h. An extract with acetonitrile was prepared, and the concentration of retinol that was in the extract was determined by an high-performance liquid chromatography (HPLC) using UV properties comparing the HM aliquots with standard solutions.

The human milk oligosaccharides (HMOs) were analyzed by employing targeted liquid chromatography mass spectrometry (LC-MS)/MS using a validated method as essentially described by Siziba et al., 2021 [31]. The quantitative determination of the HMO concentrations could be performed for the 16 most abundant HMOs and lactose: 2′-fucosyllactose (2′-FL), 3-fucosyllactose (3-FL), 3′-sialyllactose (3′-SL), 4′-galactosyllactose (4′-GL), 6′-galactosyllactose (6′-GL), 3,2′-difucosyllactose (DFL), 6′-sialyllactose (6′-SL), lacto-N-tetraose (LNT), lac-to-N-neotetraose (LNnT), lacto-N-fucopentaose-I (LNFP I), lacto-N-fucopentaose-II (LNFP II), lacto-N-fucopentaose-III (LNFP III), lacto-N-fucopentaose-V (LNFP V), lacto-N-difucohexaose I (LNDFH I), and the sum of the co-eluting lacto-N-difucohexaose II and lacto-N-neodifucohexaose II (LNDFH II + LNnDFH II). The determination of human milk types was based on the presence of specific HMO markers. Precisely, the samples were assigned to HM-type II if LNFP I and LNDFH I were below the lower limit of quantification (LLOQ). HM-type III was assigned to a sample if LNFP II and LNDFH I were below the LLOQ. HM-type IV was assigned to a sample if LNFP I, LNFP II, and LNDFH I were below the LLOQ. Finally, all of the residual HM samples were categorized as belonging to HM-type I. The Simpson’s Diversity index of the HMOs was calculated as the reciprocal sum of the square of the relative abundance of each of the measured HMOs.

### 2.4. Laboratory Procedures for Blood Analyses

Blood was drawn using an EDTA tube, and the hemogram was determined as follows: the complete cell blood counts (CBC) and hemoglobin were analyzed using Horiba’s Yumizen 500 and Pentra XLR. The hemoglobin was dosed after the red cells’ lysis. The plasma ferritin analyses were performed using BioMérieux’ multiparametric VIDAS. The plasmatic CRP and albumin analyses were performed using Horiba’s Pentra 400. Iron deficiency was defined when the plasmatic ferritin levels were <70 µg/L in case of inflammation (CRP ≥ 5 mg/L) and when the ferritin levels were <15 µg/L in the absence of inflammation (CRP < 5 mg/L) [32].

For the vitamin assessment, blood was drawn into a lithium-heparin tube and was immediately centrifuged for 15 min at 3000 r/min at 4 °C. For vitamin A and vitamin E, 100 µL of serum were stored in a cryotube at −80 °C at the IPB before being transferred to the service of Biochemistry of the Cochin Hospital in Paris (France) within 2 months. There, the vitamin A and vitamin E levels were determined using HPLC Ultimate 3000 (Thermo Scientific, Waltham, MA, USA) through a HPLC inverse phase and UV detection methods. For the vitamin C assessment, 200 µL of plasma were dissolved into 200 µL of a deproteinization solution of 2 g of meta-phosphoric acid and 15 mL 0.1% EDTA. This was vortexed for 1 min, incubated for 10 min at 4 °C, and then centrifuged for 4 min at 10,000 r/min at 4 °C. Then, the mix was stored at −80 °C until its transfer to the Cochin Hospital, where the vitamin C levels were analyzed using HPLC Ultimate 3000 (Thermo Scientific) through a HPLC inverse phase and UV detection methods at the Biochemistry service. Vitamin A deficiency was defined when the vitamin A levels were <1 µmol/L, and vitamin E deficiency was defined when the vitamin E levels were <11.6 µmol/L. Vitamin C deficiency was defined when the vitamin C levels were <11 µmol/L, according to the WHO definitions [33].

### 2.5. Statistical Analyses

The questionnaires’ data were gathered on the field using REDCap [31,34] electronic data tools that were hosted at Institut Pasteur online platform. The 24-h recall was completed on paper and then translated into a database by a trained nutritionist. Univariate analyses were performed as follows: Spearman’s coefficient was used to evaluate the correlation between the continuous variables (WDDS, HFIAS, number of meals, etc.,) with the nutrient concentration in HM; the Skillings-Mack test was used to analyze the statistical significance of the evolution of the variables over time; a Mann-Whitney test was used to assess the association of the continuous variables with the bivariate variables (food-group consumption and nutrient concentration in HM, nutrient concentration in HM and undernourishment status of the women at the point of delivery, WDDS calculation depending on the evaluation method, e.g., 24-h recall or food-frequency questionnaire, etc.); a Fisher’s exact test was used to analyze the statistical significance of the variables with different categories among the groups (HFIAS groups and consumption of food groups, etc.). Mixed models with a random intercept at the mother’s levels were used to evaluate univariate and multivariate analyses of the maternal diet on the different HM nutrients (retinol, lactose, FA, and AA). Results from the 24-h recall were considered for the final multivariate analyses as they accurately reported the real diet of the women. For the multilevel models, only models with statistically significant results are shown. These statistical analyses were performed using Stata MP Software (Stata Corp, College Station, TX, USA). The statistical significance of the *p* value was set at *p* < 0.05.

## 3. Results

### 3.1. Description of the Cohort and Maternal Characteristics at Delivery

Forty-eight women were enrolled in the MITICA study between December 2017 and June 2019. Their age ranged from 15 to 39 years, and the median age was 23 years (Table 1). Five were primigravidae. At delivery, 16 of the 46 (34.8%) women with a blood test were undernourished (which was defined by albumin plasma levels <35 g/L [35]). As the analysis of the effect of maternal undernourishment at delivery on HM was one of the purposes of the article, we will focus, here, on the women for whom we conducted an albumin measurement at delivery. The iron-deficiency rate among the women for whom we had an albumin measurement was 20/46 (43.5%), which is similar to or lower than other African cohorts and WHO estimates [36,37,38,39]. On the contrary, vitamin deficiencies were highly prevalent among these women. Concretely, 23/36 (63.9%) of the women for whom we had an albumin measurement had vitamin A deficiency, 19/44 (43.2%) had vitamin C deficiency, and 5/37 (13.5%) had vitamin E deficiency. The differences in the number of women for whom we conducted a vitamin measurement are due to the lack of blood volume available for all of the analyses. Anemia rates differed significantly between the undernourished and the non-undernourished women. While only two of the thirty non-undernourished women were anemic (7.1%), six of the sixteen undernourished women were anemic (40.0%, *p* value = 0.01). The proportion of students in the non-undernourished group almost doubled that of the ones in the undernourished group (18/30 (60.0%) vs. 6/16 (37.5%)). Precisely, the proportion of women with primary, secondary, and higher education in the non-undernourished group was 3/30 (10.0%), 21/30 (70.0%), and 6/30 (20.0%), respectively. In contrast, 5/16 (31.3%) undernourished women attended only primary school and 11/16 (68.8%) had a secondary school degree. None of them had studied at a higher education level. Table 1 presents the most significant elements of the information of the cohort.

### 3.2. Food Insecurity Indexes

At the beginning of the follow-up, 47/48 (97.9%) of the women that were included in the study lived in non-food-secure households according to the Household Food Insecurity Access Scale (HFIAS). While one out of the thirty (3.3%) non-undernourished women lived in a food-secure household, 16/30 (53.3%) and 13/30 (43.3%) lived in households with moderate and severe food insecurity, respectively. None of the undernourished women lived in food-secure households. Concretely, 6/16 (37.5%) and 10/16 (62.5%) of the undernourished mothers lived in moderately food-insecure and severely food-insecure households, respectively. Neither the HFIAS nor the Household Hunger Score (HHS) evolved significantly during the follow-up.

### 3.3. Maternal Diet Characteristics

The median number of meals per day of these women was three (Inter-quartile ratio (IQR) = 2; 3) and the median number of snacks per day that they ate was 0 (IQR = 0; 1). The total number of meals per day that the mothers ate was significantly higher during the follow-up, compared to that which was recorded one week after delivery. Compared to that which was recorded one week after delivery (median = 2, IQR = 2; 3), the women had a significantly higher number of meals at the week 4 (median = 3, IQR = 3; 3, ß-coef = 0.3, *p* value = 0.03), week 11 (median = 3, IQR = 3; 4, ß-coef = 0.6, *p* value < 0.001), week 18 (median = 3, IQR = 3; 4, ß-coef = 0.6, *p* value < 0.001), and week 25 after delivery(median = 3, IQR = 3; 4, ß-coef = 0.9, *p* value < 0.001). The maternal diet of these women was monotonous according to both the food consumption questionnaire and the 24-h recall during the entire follow-up (Table S2). Nevertheless, the WDDS was significantly lower one week after delivery, compared to later periods. According to the food consumption questionnaire, compared to that which was recorded one week after delivery (median = 3, IQR = 4; 6), the WDDSs were significantly higher at the week 4 (median = 5, IQR = 4; 6, ß-coef = 0.8, *p* value = 0.01), week 11 (median = 5, IQR = 4; 6, ß-coef = 0.7, *p* value = 0.03), week 18 (median = 5, IQR = 4; 6, ß-coef = 0.9, *p* value = 0.01), and week 25 (median = 5, IQR = 4; 6, ß-coef = 1.1, *p* value = 0.001). According to the 24-h recall, the WDDS was also significantly higher at the week 25 after birth (median = 1, IQR = 1; 5, ß-coef = 0.9, *p* value = 0.04), compared to that recorded one week after delivery (median = 1, IQR = 1; 2). In parallel, the WDDS did not vary significantly during the follow-up, according to the 24-h recalls (*n* = 161). Finally, the HHS was significantly lower at the week 18 after birth (median = 0, IQR = 0; 1, ß-coef = −0.4, *p* value = 0.02), compared to that which was recorded one week after birth (median = 0, IQR = 0; 1).

Figure S1 summarizes the precise composition of the maternal diet during the follow-up. Grains, white roots, and tubers (mainly tapioca starch and cassava) were the basis of the maternal diet, and these constituted the most consumed food groups among the mothers during the entire follow-up. Meat, poultry, and fish (dried meat, dried fish, and chicken), condiments (bouillon cubes and tomato paste and chili peppers), other vegetables (okra, onion, and tomato), and sweet foods (biscuits, home-made "pancakes", also called "local doughnuts" in Figure S1) were also paramount elements of the diet of these Central-African women after they birthed a child.

The consumption of dark green, leafy vegetables (mainly *gnetum africanum* and “goussa” or “lalo”, a plant belonging to the *Amaranthaceae* family) increased significantly along the first 6 months after birthing a child, according to both the food consumption questionnaire (*p* value < 0.001) and the 24-h recall (*p* value = 0.03). The consumption of nuts and seeds was also significantly decreased 1 week after delivery, compared to that of later periods according to the food consumption questionnaire (ß-coef = −1.6, *p* value = 0.01), and the 24-h recall (ß-coef = −1.2, *p* value = 0.04). The consumption of "other vegetables" (vegetables that have not been counted as dark green, leafy vegetables or as other vitamin A-rich vegetables) increased significantly during the 6 months after delivery according to the food consumption questionnaire (*p* value = 0.01). On the contrary, eggs were less frequently consumed according to both the food consumption questionnaire (only eaten in 5/181 (2.76%)), and the 24-h recall (2/161 (1.24%), during the entire follow-up.

Seasonality was also significantly associated with the consumption of meat, poultry, and fish, and insects according to the food consumption questionnaire and the 24-h recalls. The consumption of meat, poultry, and fish was significantly higher during the dry season, compared to the rainy season, in both the food consumption questionnaire and the 24-h recalls (75/87 (86.2%) vs. 65/91 (71.4%), *p* value = 0.02 (food consumption questionnaire); 28/94 (29.7%) vs. 15/97 (15.5%) 24-h recalls, *p* value = 0.02, respectively). On the contrary, insects were significantly more frequently consumed during the rainy season, compared to the dry season, according to the food consumption questionnaire and the 24-h recalls (16/92 (17.4%) vs. 5/87 (5.8%), *p* value = 0.03; 13/97 (13.4%) vs. 2/94 (2.1%), *p* value = 0.004, respectively).

There were significant differences in diet composition between the women who were undernourished at delivery and women who were not. According to both the food consumption questionnaire and the 24-h recalls, the consumption of dark green, leafy vegetables was significantly higher among the undernourished women compared to the women who were not undernourished at delivery (*p* value = 0.01 (food consumption questionnaire), and *p* value = 0.02 (24-h recalls)). The results of the food consumption questionnaire also show that “other beverages and foods” (represented mainly by instant coffee and undefined foods) were more frequently consumed by the undernourished women, compared to the non-undernourished women (*p* value < 0.001). The twenty-four-hour recalls displayed a significantly higher consumption of red-palm oil and sweet beverages among the undernourished women at delivery, compared to the non-undernourished mothers (*p* value = 0.01 and *p* value = 0.04, respectively). On the contrary, the consumption of powdered milk and dairy products was significantly higher among the non-undernourished women at delivery (*p* value = 0.004), according to the 24-h recalls. Further differences in diet depending on the undernourishment status of the women at delivery, the collection support tool (food consumption or 24-h recalls), and the lactation period are presented in Table S2.

### 3.4. Determinants of Lactose Levels and HM Oligosaccharides

Lactose, a disaccharide, is the sugar with the highest concentration in HM. Its concentration increased significantly during the follow-up (*p* < 0.001), according to the Skillings-Mack test. In the multivariate multilevel analyses, meat and fish consumption was associated with lower levels of lactose (aß-coef = −15.6, *p* value = 0.01), adjusted on the infant’s age. Compared to food-secure households, the women from households with moderate food insecurity had significantly higher levels of lactose (aß-coef = 3.3, *p* value = 0.02). On the contrary, food insecurity was significantly associated with reduced levels of 3-FL (severe hunger, aß-coef = −0.3, *p* value = 0.048), 6′-SL (moderate hunger, aß-coef = −0.1, *p* value = 0.01), LNT (moderate hunger, aß-coef = −0.4, *p* value = 0.01), LNnT (mild food insecurity, aß-coef = −0.1, *p* value = 0.03), LNFP-V (moderate hunger, aß-coef = −0.02, *p* value = 0.046), and LNDFH-I (mild food insecurity, aß-coef = −0.1, *p* value = 0.03) (Table 2, Table 3 and Table 4). The HHS index was also inversely associated with 2′FL levels (aß-coef = −0.2, *p* value = 0.03) in the multilevel models’ analyses.

The consumption of meat, poultry, and fish was associated with higher levels of the sum of the HMOs that were measured (aß-coef = 2.9, *p* value = 0.02), 6′-SL (aß-coef = 0.3, *p* value < 0.001), LNFP-II (aß-coef = 0.9, *p* value < 0.001), LNFP-V (aß-coef = 0.1, *p* value = 0.001), and LNDFH-II + LNnDFH-II (aß-coef = 0.4, *p* value < 0.001).

### 3.5. Dietary Determinants of HM Retinol

A high HHS was significantly associated with lower levels of retinol in the HM (aß-coef = −0.2, *p* value = 0.04), adjusted on the infant’s age. Indeed, retinol levels diminished significantly during the follow-up (*p* value < 0.001) according to the Skillings-Mack test.

### 3.6. Dietary Determinants of HM Fatty Acids Levels

The human milk of the women with moderate food insecurity had significantly lower levels of total fatty acids, compared to the women who lived in food-secure households (aß-coef = −7.2, *p* value = 0.03). More precisely, some degree of food insecurity was significantly associated with lower HM levels of Omega-3 poly-unsaturated fatty acids (PUFAs) (absolute and relative levels: mild food insecurity aß-coef = −275.8, *p* value = 0.04; mild food insecurity aß-coef = −0.7, *p* value = 0.002, respectively), docosahexaenoic acid (DHA) (absolute and relative levels: moderate food insecurity aß-coef = −34.9, *p* value = 0.04; mild food insecurity aß-coef = −0.4, *p* value = <0.001, respectively), and eicosapentaenoic acid (EPA) (absolute and relative levels: mild food insecurity aß-coef = −55.1, *p* value = 0.003, moderate food insecurity aß-coef = −16.0, *p* value = 0.01; moderate food insecurity aß-coef = −0.03, *p* value = 0.04, respectively). Fish was the food group that was most frequently associated with higher levels of HM fatty acids. Concretely, fish consumption was statistically linked with higher HM levels of PUFAs (relative levels: aß-coef = 1.0, *p* value = 0.047), Omega-3 PUFAs (relative levels: aß-coef = 0.2, *p* value = 0.01), arachidonic acid (ARA) (relative levels: aß-coef = 0.04, *p* value = 0.01), DHA (absolute and relative levels: aß-coef = 43.7, *p* value = 0.01, and aß-coef = 0.1, *p* value = <0.001, respectively); EPA (absolute and relative levels: aß-coef = 15.3, *p* value = 0.01, and aß-coef = 0.04, *p* value = 0.01, respectively). More globally, the consumption of the food categories “meat, poultry, and fish”, “insects and small rodents” (including insect larvae/grubs, insect eggs, fish roe, spiders, land and sea snails, and any other small invertebrates [22]), “other vegetables”, and “other oils and fats” were also significantly associated with higher levels of different fatty acids in HM (Table 3). The food group “other oils and fats” comprises mainly peanut oil, but also lard, suet (tallow), and butter (solid animal fats); margarine and hydrogenated vegetable oil; a range of oils that have been extracted from nuts, seeds, and grains [22]. Further dietary determinants of HM fatty acids levels are presented in Table 5 and Table 6. Only the multilevel models with significant results are shown.

### 3.7. Dietary Determinants of Amino Acids

#### 3.7.1. Total Amino Acids

Moderate hunger in the household (according to the HHS), compared to “no to little hunger in the household”, was significantly associated with low levels of each of the amino acids that were analyzed in the study (Table 7 and Table 8). Arginine levels were also inversely correlated with the HHS (aß-coef = −26.7, *p* value = 0.03). An elevated consumption of meat, poultry, and fish, and red palm oil was also significantly associated with higher levels of HM amino acids (aß-coef = 5484.4, *p* value < 0.001; aß-coef = 2825.1, *p* value = 0.003, respectively). On the contrary, the consumption of nuts (tree nuts but also groundnuts (peanuts), certain seeds, and seed butters, such as pounded groundnut/peanut butter or peanut paste, cashew butter, or sesame butter (tahini) when it was consumed in doses that were bigger than 15 g) (aß-coef = −4798.2, *p* value < 0.001), other vegetables (aß-coef = − 1205.7, *p* value = 0.03), insects (aß-coef = −1209.9, *p* value = 0.03), and condiments (aß-coef = −842.6, *p* value = 0.01) were inversely associated with the HM amino acid levels. Indeed, these dietary determinants of HM amino acids were almost unanimously homogeneous among the different amino acids (Table 7 and Table 8). Additionally, the concentration of total amino acids in HM decreased significantly during the follow-up. Compared to the HM one week after birth, the total content of all of the amino acids diminished significantly and progressively for 25 weeks.

Increased food insecurity indexes were associated with lower relative values of aspartic acid and asparagine (aß-coef = −0.01, *p* value = 0.049), histidine (aß-coef = −0.1, *p* value = 0.002), and phenylalanine (aß-coef = −0.1, *p* value = 0.03).

#### 3.7.2. Free Amino Acids

Human milk free amino acids were also significantly influenced by food insecurity levels. Precisely, compared to the category “no hunger in the household”, women who lived in households with “moderate hunger in the household” (according to HHS) had significantly lower levels of total free amino acids in HM (aß-coef = −62.8, *p* value = 0.04). Moreover, the indexes of food insecurity were significantly correlated with lower levels of free arginine (aß-coef = −0.1, *p* value = 0.02), asparagine (aß-coef = −1.3, *p* value < 0.001), histidine (aß-coef = −1.4, *p* value = 0.03), lysine (aß-coef = −0.7, *p* value = 0.04), taurine (aß-coef = −7.9, *p* value = 0.03), threonine (aß-coef = −4.9, *p* value = 0.002), tryptophan (aß-coef = −0.04, *p* value = 0.04), tyrosine (aß-coef = −1.8, *p* value = 0.02), and valine (aß-coef = −3.0, *p* value = 0.03).

The consumption of pulses was also associated with higher levels of the sum of the free amino acids (aß-coef = 116.6, *p* value = 0.04), alanine (aß-coef = 16.3, *p* value = 0.003), lysine (aß-coef = 3.9, *P* value = 0.01), and threonine (aß-coef = 7.5, *p* value = 0.01). Other dietary elements that were associated with the free amino acid concentration in the breastmilk are detailed in Table 9 and Table 10.

## 4. Discussion

The MITICA study is one of the only studies linking HM composition, maternal diet, and maternal nutritional status at delivery in Central-Africa. We found a high food insecurity burden in addition to a low diverse maternal diet that was based on grains, tubers, and white roots. Even in the context of extended food insecurity in our cohort, high household food insecurity levels were significantly associated with reduced levels of fatty acids, total amino acids, free amino acids, retinol, and up to seven different HMOs in the HM. This is particularly worrisome as the “breastfeeding paradox” shows that the households with an increased risk of food insecurity tend to reduce breastfeeding in quantity and duration in Western and African countries [40,41,42,43,44,45]. Hence, adverse consequences may arise in the infants that live in these already vulnerable households. Unfortunately, further evidence on the influence of food insecurity on HM nutritional content is extremely limited. Yet, a recent study showed that the percentage of energy from both carbohydrates and fats in the maternal diet was significantly associated with the HM total energy [46]. Some authors have postulated that HM composition might rather be related with maternal body composition [47]. Indeed, in samples that were taken at the first (*n* = 40), third (*n* = 22), and sixth (*n* = 15) lactation months, their nutrient intake was not correlated with the HM composition among Polish women, but the variance in milk fat was significantly correlated with the body mass index (BMI) in the first month postpartum, thereby underling the association of maternal body composition with the nutritional content of HM [47]. In any case, it is reasonable to consider that diet is one of the major determinants of maternal body composition. In our study, the maternal diet differed significantly between undernourished and non-undernourished women at delivery. Undernourished women at delivery were significantly more likely to consume dark green, leafy vegetables, red palm oil, and sweet beverages, whereas milk and dairy product consumption prevailed among the non-undernourished women. In parallel, the food insecurity indexes were also significantly associated with the undernourishment status of the women at delivery.

In the broadest review articles investigating the influence of maternal diet on HM composition, the maternal dietary intakes of FA and some micronutrients—including fat soluble vitamins, vitamin B1, and vitamin C—were significantly associated with their concentration in HM [48,49,50,51]. Concretely, in the majority of studies FAs in the maternal diet are, with some exceptions [52,53], positively associated with FAs in HM [11,13,54,55,56,57,58,59,60,61,62,63,64,65,66,67,68,69,70]. Also, interventional studies have shown that supplementation with FAs resulted in a significant increase of FAs in HM [71,72]. Furthermore, studies from very different countries, worldwide, have shown that lactating women who consume fish and other foods with high PUFA levels display relatively higher HM fatty acids, especially DHA [62,73,74,75,76]. In our study, fish consumption was also significantly associated with higher levels of FAs in HM (Table 5). Further, the consumption of “fats and oils”, “other vegetables”, and “insects and small rodents” was also associated with higher FA levels in HM. These results are also coherent with the previous literature [11,51,77,78]. However, the FA levels in HM were lower than they were in pre-existing cohorts [79]. Additionally, the total FA concentration in HM remained constant during the first 6 months of the infant’s life, which is in contrast with other cohorts [6,80]. In sum, dietary determinants of HM FAs do not differ significantly from other cohorts despite the low FAs levels in HM and the high rates of food insecurity. Nevertheless, the meaningful effect of food insecurity on HM FAs is likely fundamental to explaining the low levels of FAs that are in this cohort, compared to those of others cohorts. Indeed, these low levels of FAs cannot be ascribed solely to the ethnic variations in HM FA content that have already been observed in other populations, including in African cohorts [11,81]. Therefore, the effect of food insecurity on HM FA composition may be greater than it has been previously postulated.

In parallel to the FAs and compared to the category “no hunger in the household”, a “moderate level of hunger in the household” was significantly associated with reduced levels of both free amino acids and the total level of amino acids. While certain studies suggest that there is a lack of evidence associating maternal diet with HM amino acids [47,48], the dietary determinants of the total amino acids were homogeneous among the different amino acids that were analyzed in our study. Precisely, we found that “meat, poultry, and fish”, and red-palm oil consumption was associated with high levels of total amino acids [82]. In previous research, protein intake has also been associated with higher amino acid levels in HM [48,50]. On the contrary, in our cohort, women who reported a high consumption of “nuts”, “other vegetables”, and “insects and small rodents” displayed significantly lower levels of total amino acids in their HM. Some studies report a positive correlation between maternal egg intake and amino acid levels in HM [82,83]. The effect of egg intake on amino acids’ levels in HM could not be properly assessed in our cohort as egg consumption was extremely rare among the women in Bangui (only five eggs were reported to be eaten the previous day during the entire follow-up). Further limitations of the study include the limited sample-size, the lack of a homogeneous schedule for the foremilk sampling (sampling time fluctuated from 10 to 12 AM), and the limitation of the food intake assessment to the 24-h preceding the sampling.

The effect of diet on HMO composition has been seldom analyzed as it is an emerging research field and HM types are genetically influenced. More concretely, in a 2021 survey on HM determinants, only three studies considered the maternal diet [84]. Further, some authors have reported no significant association of maternal diet with HMO levels [85,86,87]. Azad et al. found that, independent of the secretor status and lactation stage, seasonal variation, geographic location, parity, ethnicity, and exclusive breastfeeding were significant determinants of some HMO levels in the CHILD Canadian cohort [85]. On the contrary, diet quality and the mode of delivery were not significantly associated with the HMOs analyzed in the CHILD cohort study. However, in this same cohort, maternal diet and body mass were interrelated and associated with HM microbiota [88]. In parallel, in another study including both Swedish and Gambian women, some HMOs were significantly associated with maternal age, postpartum period, weight, and body-mass index [89]. Further, the HMOs from ethnically similar populations varied geographically, suggesting that HMO levels might also be influenced by the environment.

In our cohort, in parallel to the rest of nutrients, a large number of HMOs were significantly associated with food insecurity levels. In Bangladesh, the mothers of undernourished children also showed significantly lower levels of HMOs, even if neither the maternal undernourishment status nor the maternal diet were assessed [90].

Here, meat and poultry consumption were associated with higher HMO levels. Other studies have also shown similar results. Qiao et al. showed that a higher dietary intake of milk, beef, egg, mutton, and pork was associated with higher milk sialic acid levels [91]. Recent research has demonstrated that maternal dietary carbohydrate and energy sources alter HMO concentrations significantly, including fucosylated species [92]: fucose and galactose might be recycled by specific monosaccharide metabolic pathways that are in mammalian cells [93,94]. Furthermore, the previous study reveals that this dynamic process, by which maternal diet modifies the HMO composition during lactation, also modulates the HM-associated microbiota [92]. In the context of maternal undernutrition, this might entail meaningful differences in the infant oral and gastrointestinal bacterial colonization, thereby resulting in an impaired metabolism and immune development [95,96,97,98,99,100]. HMOs provide fucose and sialic acid which are essential for brain development [101,102]. Sialic acid also plays a significant role in the formation of synapses and its concentration in HM is influenced by the maternal diet [103,104,105,106]. Therefore, maternal food insecurity might have long-term effects with long-lasting consequences for the child. However, evidence on how food insecurity or maternal undernourishment might influence lactose levels (inversely, compared to the rest of HM nutrients) remains a topic for further research.

Maternal diets modulates the maternally secreted micro-RNAs (miRNAs) in HM that are stable in HM fat globules [107]. These miRNAs are involved in DNA methylation, histone modification, and chromatin remodeling and might have important regulatory functions in the infant’s development and metabolism, such as FTO, INS, and IGF1 modulation [108,109,110,111,112], in addition to their essential immune properties [113,114]. Moreover, recent studies have described the meaningful role of miRNAs in neurodevelopment [112,115,116,117]. Indeed, they constitute a substantial part of the health benefits of HM [5], and they might be reduced due to the low levels of fat in the cohort. The effect of low fat levels in HM on the infant’s metabolism and neurodevelopment needs to be assessed in prospective cohorts.

## 5. Conclusions

Food insecurity and maternal diet, via nutrient intake reduction in HM, might exert a considerable impact on the infant’s undernourishment risk.

Beyond the direct effect of nutrient deficiencies in HM, epigenetic alterations affecting the infant’s metabolism and development might arise in the context of maternal undernourishment.

Nutritional alterations in HM—especially in HMO—might alter the physiological assembly of the gut microbiota of the infant, thereby resulting in an impaired immune priming and metabolic function [95,96,97,98,99,100]. Infant gut colonization should be investigated in prospective clinical follow-ups in the context of altered HM composition to assess its consequences.

In conclusion, our results plead for consistent actions on food security as an effective manner to influence the nutritional content of HM and thereby, potentially improve the infants’ survival and healthy growth. Human milk is the most unique nutritional source for infants, therefore, food security, maternal nutritional status, and maternal dietary factors might entail founding effects and diverse trajectories for the infant’s growth and their immune and neurocognitive development. In parallel, the pathological pathways of maternal undernourishment and its influence on HM biosynthesis remain deeply elusive. Supplementary research to unravel the molecular mechanisms that are responsible for the fluctuations in HM nutrient concentration in the context of maternal undernutrition remain, therefore, of paramount importance.

## Figures and Tables

**Table 1:** Baseline characteristics of the women that were included in the MITICA study for whom we had an albumin measurement at delivery.

	All Women	Not Undernourished Women *	Undernourished Women *	*p* Value **
Number of participants	46	30 (65.2%)	16 (34.8%)	**0.04**
Age (years)	22.5 (20.6; 29.5)	22.4 (19.7; 29.8)	22.7 (21.3; 29.4)	0.7
Gravidity	1 (1; 3)	1 (1; 2)	2 (1; 3)	0.2
Primigravidae	5 (11.1%)	3 (10.3%)	2 (12.5%)	reference
1–3 previous gestations	36 (80.0%)	23 (86.2%)	11 (68.8%)	0.7
4+	4 (8.9%)	1 (3.5%)	3 (18.8%)	0.3
Education				0.1
Primary	8 (17.4%)	3 (10.0%)	5 (31.3%)	reference
Secondary	32 (69.6%)	21 (70.0%)	11 (68.8%)	0.2
Higher	6 (13.0%)	6 (20.0%)	0	
Occupation				0.2
Homecare	21 (45.7%)	11 (36.7%)	10 (62.5%)	reference
Work outside home	1 (2.2%)	1 (3.3%)	0	
Student	24 (52.2%)	18 (60.0%)	6 (37.5%)	0.2
Weight (kg)	63.5 (60.0; 67.0)	63.5 (60.0; 67.0)	63.0 (58.5; 67.5)	0.8
Height (m)	1.6 (1.6; 1.7)	1.6 (1.6; 1.7)	1.61 (1.5; 1.6)	0.8
Fever (T > 38 C)	3 (6.5%)	2 (6.7%)	1 (6.3%)	0.9
Albumin (g/L)	36.0 (33.0; 38.0)	38.0 (36.0; 40.0)	32.0 (31.0; 33.0)	**<0.001**
Hemoglobin (g/dL)	12.4 (11.2; 13.8)	12.5 (11.6; 13.6)	11.3 (10.0; 14.1)	0.2
Anemia (Hemoglobin < 11.0 g/dL)	8 (18.6%)	2 (7.1%)	6 (40.0%)	**0.01**
Ferritin (ng/mL)	48.0 (27.0; 105.0)	52.5 (35.0; 108.0)	29.5 (21.0; 69.0)	0.2
Iron deficiency ***	20 (43.5%)	12 (40.0%)	8 (50.0%)	0.5
Vitamin C plasmatic levels (µmol/L)	15.0 (5.7; 29.5)	20.4 (8.2; 36.9)	10.2 (3.7; 18.7)	0.1
Vitamin C deficiency (vitamin C levels < 11 µmol/L)	19 (43.1%)	10 (35.7%)	9 (56.3%)	0.2
Vitamin A seric levels (µmol/L)	0.9 (0.8; 1.1)	0.9 (0.8; 1.1)	0.9 (0.8; 1.3)	0.9
Vitamin A deficiency (vitamin A seric levels < 1 µmol/L)	23 (63.9%)	14 (63.6%)	9 (64.3%)	0.9
Vitamin E seric levels (µmol/L)	26.0 (20.6; 30.1)	26.8 (20.4; 30.8)	24.9 (20.6; 29.5)	0.6
Vitamin E deficiency (vitamin E levels < 11.6 µmol/L)	5 (13.5%)	4 (17.4%)	1 (7.1%)	0.6
CRP (mg/L)	7.0 (1.8; 26.2)	6.2 (1.6; 14.4)	20.5 (2.3; 29.1)	0.2
Inflammation (CRP > 5 mg/L)	28 (60.9%)	18 (60.0%)	10 (62.5%)	0.9

* Undernourished status was defined by albumin levels <35 g/L. ** *p* value of the difference between undernourished and non-undernourished women (numbers in bold). *** Iron deficiency was defined by serum ferritin levels < 15 ng/mL in the absence of inflammation. In the case of inflammation, iron deficiency was defined by ferritin levels < 70 ng/mL. For the continuous variables, median and inter-quartile range are provided. Mann-Whitney test was used to evaluate the difference in these variables among undernourished and non-undernourished women. For the binary variables and variables with categories, n and percentages are provided. Fisher’s exact test was used to evaluate the difference in these variables among undernourished and non-undernourished women.

**Table 2:** Multilevel models of the dietary determinants of human milk oligosaccharide levels during the first 6 months of life (1).

	Total HMOs (g/L)	*p* Value	2′FL (g/L)	*p* Value	3−FL (g/L)	*p* Value	3′SL (g/L)	*p* Value	6′−SL (g/L)	*p* Value
Woman’s dietary diversity score;			0.2 (−0.0; 0.5)	0.05						
Nuts and seeds;									−0.2 (−0.3; −0.1)	0.001
Meat, poultry, and fish;	2.9 (0.5; 5.2)	0.02							0.3 (0.1; 0.4)	<0.001
Other vitamin A-rich fruits and vegetables;							0.1 (0.1; 0.2)	<0.001		
Other vegetables;			−0.6 (−1.1; −0.1)	0.01						
Condiments and seasonings;					−0.1 (−0.1; −0.0)	0.04				
Fish;			0.3 (0.1; 0.6)	0.004	−0.1 (−0.1; −0.0)	0.03				
Household hunger scale index;			−0.2 (−0.3; −0.0)	0.03						
HHS: Little to no hunger;					reference				reference	
Moderate hunger;					0.1 (−0.0; 0.2)	0.1			−0.1 (−0.1; −0.0)	0.01
Severe hunger;					−0.3 (−0.7; −0.0)	0.048			0.0 (−0.1; 0.2)	0.8
Milk-type group I;	−0.2 (−0.9; 0.5)	0.5	−2.0 (−1.9; −0.5)	0.001	0.4 (0.0; 0.5)	<0.001	0.0 (−0.0; 0.0)	0.8	−0.0 (−0.1; 0.0)	0.3
Milk-type group II;	−1.5 (−2.3; −0.7)	<0.001	−3.2 (−4.0; −2.3)	<0.001	1.2 (1.1; 1.3)	<0.001	0.0 (−0.0; 0.0)	0.9	−0.1 (−0.1; −0.0)	0.02
Milk-type group III;	reference		reference		reference		reference		reference	
Milk-type group IV;	−2.5 (−3.5; −1.6)	<0.001	−3.2 (−4.3; −2.2)	<0.001	−0.0 (−0.1; 0.1)	0.9	0.1 (0.0; 0.1)	<0.001	0.0 (−0.0; 0.1)	0.5
Undernourished mother at delivery			−0.6 (−1.3; −0.03)	0.04						

**Table 3:** Multilevel models of the dietary determinants of human milk oligosaccharide levels during the first 6 months of life (2).

	6′−GL (g/L)	*p* Value	LNT (g/L)	*p* Value	LNnT (g/L)	*p* Value	LNFP−I (g/L)	*p* Value	LNFP−II (g/L)	*p* Value
Nuts and seeds;	−0.0 (−0.0; −0.0)	0.04								
Meat, poultry, and fish;									0.9 (0.5; 1.3)	<0.001
Dark green leafy vegetables;			0.4 (0.1; 0.6)	0.01						
Other oils and fats;			0.2 (0.0; 0.4)	0.046						
Condiments and seasonings;			0.3 (0.1; 0.4)	0.001						
Other beverages and foods;			−1.2 (−2.1; −0.4)	0.004						
Sweet foods;			0.8 (0.0; 1.6)	0.04						
Red palm oil;					−0.1 (−0.1; −0.0)	0.03				
HFIAS: Food security;					reference					
Mildly food insecure;					−0.1 (−0.1; −0.0)	0.03				
Moderately food insecure;					−0.0 (−0.0; 0.0)	0.7				
HHS: Little to no hunger;			reference							
Moderate hunger;			−0.4 (−0.7; −0.1)	0.01						
Severe hunger;			0.8 (−0.3; 1.8)	0.1						
Milk-type group I;			0.3 (−0.0; 0.7)	0.05			−0.5 (−0.7; −0.3)	<0.001	0.3 (0.2; 0.4)	<0.001
Milk-type group II;			0.6 (0.2; 1.0)	0.002			−0.8 (−1.0; −0.6)	<0.001	0.7 (0.6; 0.9)	<0.001
Milk-type group III;			reference				reference		reference	
Milk-type group IV;			1.7 (1.2; 2.1)	<0.001			−0.9 (−1.1; −0.6)	<0.001	0.0 (−0.1; 0.2)	0.81
Female infant;	0.0 (0.0; 0.1)	0.01	0.3 (0.1; 0.6)	0.02					0.1 (0.1; 0.2)	0.001
Undernourished mother at delivery			0.4 (0.1; 0.7)	0.01						

**Table 4:** Multilevel models of the dietary determinants of human milk oligosaccharide levels during the first 6 months of life (3).

	LNFP−III (g/L)	*p* Value	LNFP−V (g/L)	*p* Value	LNDFH−I (g/L)	*p* Value	LNDFH−II + LNnDFH−II (g/L)	*p* Value
Grains, white roots, and tubers;					−0.1 (−0.1; −0.0)	0.02		
Meat, poultry, and fish;			0.1 (0.0; 0.1)	0.001			0.4 (0.3; 0.4)	<0.001
Other vegetables;					0.1 (0.0; 0.2)	0.01		
Other fruits;					−0.2 (−0.4; −0.0)	0.02		
Insects, small rodents, and other small animals;					−0.1 (−0.2; −0.0)	0.001		
Red palm oil;	−0.1 (−0.1; −0.0)	0.03						
Other oils and fats;					−0.1 (−0.2; −0.1)	<0.001		
Condiments and seasonings;	−0.0 (−0.1; −0.0)	0.02						
HFIAS: Food security;					reference			
Mildly food insecure;					−0.1 (−0.3; −0.0)	0.03		
Moderately food insecure;					−0.0 (−0.1; 0.0)	0.7		
HHS: Little to no hunger;			reference					
Moderate hunger;			−0.0 (−0.0; −0.0)	0.046				
Severe hunger;			0.0 (−0.0; 0.1)	0.9				
Milk-type group I;			0.0 (0.0; 0.0)	<0.001	0.4 (0.3; 0.4)	<0.001	0.0 (0.0; 0.0)	0.02
Milk-type group II;			0.1 (0.1; 0.1)	<0.001	0.0 (−0.1; 0.1)	0.6	0.1 (0.1; 0.1)	<0.001
Milk-type group III;			reference		reference		reference	
Milk-type group IV;			0.0 (−0.0; 0.0)	0.1	0.0 (−0.1; 0.1)	0.6	−0.0 (−0.0; 0.0)	0.9
Female infant;			0.0 (0.0; 0.0)	<0.001				

Multilevel models with an intercept at the mother’s level were adjusted on the infant’s age. Adjusted beta-coefficients of the different variables are given with their 95% CI in parentheses. HFIAS: Household food insecurity access scale. HHS: Household hunger scale index.

**Table 5:** Multilevel models of the dietary determinants of human milk fatty acids during the first 6 months of life (1).

	Total Fatty Acids (g/L)	*p* Value	PUFAs (%)	*p* Value	Omega-6 PUFAs (%)	*p* Value	Omega-3 PUFAs (mg/L)	*p* Value	Omega-3 PUFAs (%)	*p* Value
Grains and tubers;							−143.5 (−251.2; −35.7)	0.01		
Pulses;										
Meat, poultry, and fish;	30.0 (−0.5; 60.6)	0.05								
Other vegetables;			1.9 (0.2; 3.7)	0.03	1.9 (0.1; 3.7)	0.04			0.5 (0.2; 0.8)	0.002
Insects and small rodents;									0.6 (0.3; 0.9)	<0.001
Other oils and fats;									0.3 (0.1; 0.5)	0.001
Condiments and seasonings;			−1.3 (−2.3; −0.2)	0.02	−1.2 (−2.3; −0.2)	0.02			0.1 (0.1; 0.5)	<0.001
Fish (in any form);			1.0 (0.0; 2.0)	0.047					0.2 (0.1; 0.3)	0.01
HFIAS: Food security;	reference						reference		reference	
Mildly food insecure;	−6.0 (−27.3; 15.4)	0.58					−275.8 (−539.8; −11.9)	0.04	−0.7 (−1.1; −0.2)	0.002
Moderately food insecure.	−7.2 (−13.9; −0.6)	0.03					−78.9 (−162.3; 4.4)	0.06	−0.1 (−0.2; 0.0)	0.2
	**MUFAs (%)**	***p* Value**	**SFAs (%)**	***p* Value**	**LA (%)**	***p* Value**	**ARA (mg/L)**	***p* Value**	**ARA (%)**	***p* Value**
Grains and tubers;										
Pulses;	−4.3 (−8.4; −0.2)	0.04								
Nuts and seeds;	−8.2 (−14.9; −1.6)	0.02	9.6 (0.1; 19.1)	0.048	−5.6 (−10.6; −0.6)	0.03				
Meat, poultry, and fish;							120.8 (6.6; 232.9)	0.04		
Other vitamin A-rich fruits and vegetables;	−3.9 (−7.8; −0.1)	0.04	5.9 (0.4; 11.3)	0.03	−3.5 (−6.4; −0.6)	0.02				
Other vegetables;					2.1 (0.4; 3.8)	0.01				
Condiments and seasonings;					−1.5 (−2.5; −0.5)	0.003				
Fish (in any form);									0.0 (0.0; 0.1)	0.01
Undernourished mother at delivery					1.3 (0.2; 2.4)	0.02			−0.1 (−0.1; −0.0)	0.01

Multilevel models with an intercept at the mother’s level were adjusted on the infant’s age. Adjusted beta-coefficients of the different variables are given with their 95% CI in parentheses. HFIAS: Household food insecurity access scale.

**Table 6:** Multilevel models of the dietary determinants of human milk fatty acids during the first 6 months of life (2).

	DHA (mg/L)	*p* Value	DHA (%)	*p* Value	ALA (mg/L)	*p* Value	EPA (mg/L)	*p* Value	EPA (%)	*p* Value
Grains and tubers									−0.1 (−0.1; −0.0)	<0.001
Other vegetables	100.3 (26.9; 173.8)	0.01								
Insects and small rodents			0.2 (0.0; 0.3)	0.04	109.9 (39.5; 180.3)	0.002				
Other oils and fats			0.2 (0.1; 0.3)	0.002						
Condiments and seasonings			0.1 (0.0; 0.2)	0.047	42.6 (2.8; 82.4)	0.04				
Fish (in any form)	43.7 (10.3; 77.2)	0.01	0.1 (0.1; 0.2)	<0.001			15.3 (3.1; 27.6)	0.01	0.0 (0.0; 0.1)	0.01
HFIAS: Food security	reference		reference				reference		reference	
Mildly food insecure	−97.7 (−202.4; 7.0)	0.07	−0.4 (−0.7; −0.2)	<0.001			−55.1 (−91.4; −18.8)	0.003	−0.1 (−0.2; 0.0)	0.06
Moderately food insecure	−34.9 (−67.4; −2.27)	0.04	−0.0 (−0.1; 0.0)	0.2			−16.0 (−28.2; −3.9)	0.01	−0.0 (−0.1; −0.0)	0.04

Multilevel models with an intercept at the mother’s level were adjusted on the infant’s age. Adjusted beta-coefficients of the different variables are given with their 95% CI in parentheses. HFIAS: Household food insecurity access scale.

**Table 7:** Multilevel models of the dietary determinants of human milk total amino acids during the first 6 months of life (1).

	Alanine (µg/mL)	*p* Value	Arginine (µg/mL)	*p* Value	Glycine (µg/mL)	*p* Value	Histidine (µg/mL)	*p* Value
Nuts and seeds	−244.8 (−419.3; −70.3)	0.01	−263.9 (−476.5; −51.2)	0.02	−154.1 (−280.2; −28.2)	0.02	−140.0 (−226.1; −54.1)	0.001
Meat, poultry, and fish	317.4 (132.9; 501.8)	0.001	312.4 (97.4; 527.4)	0.004	238. 6 (105.7; 371.5)	<0.001	152.5 (62.8; 242.1)	0.001
Other vegetables							−40.4 (−75.7; −5.1)	0.03
Insects, small rodents, and other small animals			−110.1 (−193.3; −26.9)	0.01				
Red palm oil	237.9 (114.3; 361.6)	<0.001	201.2 (45.2; 357.1)	0.01	185.6 (96.2; 275.1)	<0.001	118.0 (56.8; 179.3)	<0.001
Condiments and seasonings			−61.9 (−112.1; −11.7)	0.02			−19.1 (−38.0; −0.2)	0.047
Household hunger scale index			−26.7 (−50.7; −2.6)	0.03				
HHS: Little to no hunger	reference				reference		reference	
Moderate hunger	−88.1 (−152.2; −24.0)	0.01			−52.8 (−98.9; −6.6)	0.03	−64.3 (−96.1; −32.4)	<0.001
Severe hunger	22.4 (−184. 0; 228.8)	0.8			−0.7 (−148.2; 146.9)	0.9	22.0 (−81.0; 125.0)	0.7
Undernourished mother			−67.5 (−123.3; −11.6)	0.02				
	**Isoleucine (µg/mL)**	***p*** **Value**	**Leucine (µg/mL)**	***p*** **Value**	**Lysine (µg/mL)**	***p*** **Value**	**Methionine (µg/mL)**	***p*** **Value**
Nuts and seeds	−229.7 (−366.0; −93.4)	0.001	−523.4 (−803.3; −243.6)	<0.001	−398.5 (−631.1; −165.9)	0.001	−96.5 (−146.0; −47.0)	<0.001
Meat, poultry, and fish	225.0 (82.6; 367.4)	0.002	563.4 (270.3; 857.0)	<0.001	439.0 (196.4; 681.6)	<0.001	88.6 (37.1; 140.0)	0.001
Other vegetables	−64.3 (−120.6; −7.9)	0.03	−135.3 (−251.7; −18.8)	0.02	−111.5 (−207.6; −15.5)	0.02	−23.1 (−43.5; −2.7)	0.03
Insects, small rodents, and other small animals	−70.2 (−123.7; −16.6)	0.01	−168. 4 (−277.0; −59.7)	0.002	−115.9 (−207.3; −24.5)	0.01	−25.7 (−44.9; −6.6)	0.01
Red palm oil	141.4 (43.5; 239.3)	0.01			294. 5 (127.3; 461.6)	0.001		
Condiments and seasonings	−38.7 (−71.6; −5.7)	0.02	−111.2 (−176.1; −46.2)	0.001	−82.4 (−138.6; −26.3)	0.004	−18.2 (−29.6; −6.7)	0.002
HHS: Little to no hunger	reference		reference		reference		reference	
Moderate hunger	−122.6 (−174.2; −70.9)	<0.001	−259.8 (−366.3; −153.3)	<0.001	−187.9 (−275.8; −99.9)	<0.001	−45.5 (−64.1; −26.8)	<0.001
Severe hunger	54.5 (−111.9; 221.0)	0.5	86.3 (−266.4; 439.0)	0.6	43.0 (−238.9; 324.9)	0.8	13.6 (−46.0; 73.2)	0.7

Multilevel models with an intercept at the mother’s level were adjusted on the infant’s age. Adjusted beta-coefficients of the different variables are given with their 95% CI in parentheses. HHS: Household hunger scale index.

**Table 8:** Multilevel models of the dietary determinants of human milk total amino acids during the first 6 months of life (2).

	Phenylalanine (µg/mL)	*p* Value	Serine (µg/mL)	*p* Value	Taurine (µg/mL)	*p* Value	Threonine (µg/mL)	*p* Value
Nuts and seeds	−275.6 (−437.1; −114.2)	0.001					−248.7 (−434.0; −63.3)	0.01
Meat, poultry, and fish	329.2 (161.3; 497.1)	<0.001	442.9 (219.9; 665.9)	<0.001			369.0 (171.3; 566.7)	<0.001
Other vegetables	−74.8 (−141.2; −8.3)	0.03						
Insects, small rodents, and other small animals	−71.8 (−141.2; −8.3)	0.03						
Red palm oil	221.7 (105.5; 338.0)	<0.001	290.1 (141.1; 439.2)	<0.001			311.7 (180.9; 442.5)	<0.001
Condiments	−53.0 (−91.9; −14.0)	0.01						
HHS: Little to no hunger	reference		reference		reference		reference	
Moderate hunger	−115.6 (−176.7; −55.1)	<0.001	−111.2 (−187.8; −34.6)	0.004	−9.37 (−17.58; −1.16)	0.03	−102.3 (−171.1; −33.6)	0.004
Severe hunger	27.4 (−165.1; 220.0)	0.8	35.3 (−212.5; 283.1)	0.8	−4.74 (−31.48; 22.00)	0.73	17.3 (−211.3; 245. 8)	0.9
Milk-type group I			−78.2 (−152.7; −3.7)	0.04	−10.4 (−18.5; −2.3)	0.01		
Milk-type group II			−64.8 (−150.4; 20.9)	0.1	−7.2 (−16.5; 2.0)	0.1		
Milk-type group III			reference		reference			
Milk-type group IV			32.6 (−70.7; 135.9)	0.5	−0.9 (−12.2; 10.4)	0.9		
	**Tyrosine (µg/mL)**			***p* Value**	**Valine (µg/mL)**	***p*** **Value**	**Sum of the Total Amino Acids (µg/mL)**	***p*** **Value**
Nuts and seeds	−199.7 (−324.4; −74.9)			0.002	−337.9 (−524.9; −151.0)	<0.001	−4798.2 (−7427.1; −2169.3)	<0.001
Meat, poultry, and fish	287.3 (153.8; 420.7)			<0.001	365.6 (170.3; 560.9)	<0.001	5484.4 (2739.1; 8229.6)	<0.001
Other vegetables	−53.1 (−105.4; −0.9)			0.046	−94.7 (−172.0; −17.3)	0.02	−1209.9 (−2297.0; −122. 7)	0.03
Insects, small rodents and other small animals					−91.0 (−172.0; −17.5)	0.02	−1340.6 (−2374.0; −307.3)	0.01
Red palm oil	199.3 (111.7; 286.8)			<0.001	173.0 (38.8; 307.3)	0.01	2825.1 (936.3; 4713. 9)	0.003
Condiments					−56.9 (−102.0; −11.7)	0.01	−842.6 (−1477.5; −207. 6)	0.01
HHS: Little to no hunger	reference				reference		reference	
Moderate hunger	−80.9 (−128.5; −33.4)			0.001	−154.2 (−225.1; −83.4)	<0.001	−2121.0 (−3116.6; −1125.4)	<0.001
Severe hunger	40.9 (−117.2; 199.1)			0.6	74.1 (−154.4; 302.6)	0.5	596.7 (−2606.6; 3800.0)	0.7

Multilevel models with an intercept at the mother’s level were adjusted on the infant’s age. Adjusted beta-coefficients of the different variables are given with their 95% CI in parentheses. HHS: Household hunger scale index.

**Table 9:** Multilevel models of the dietary determinants of human milk free amino acids during the first 6 months of life (1).

	Alanine (µg/mL)	*p* Value	Arginine (µg/mL)	*p* Value	Asparagine (µg/mL)	*p* Value	Aspartic Acid (µg/mL)			*p* Value
Pulses: beans, peas, and lentils	16.3 (6.4; 26.2)	0.001								
Red palm oil			3.2 (1.1; 5.3)	0.003						
Fish					0.7 (0.0; 1.3)	0.049	−2.6 (−4.9; −0.2)			0.03
HFIAS			−0.1 (−0.2; −0.0)	0.02						
Food security					reference					
Mildly food insecure					0.5 (−1.4; 2.4)	0.6				
Moderately food insecure					−1.3 (−1.9; −0.7)	<0.001				
Little to no hunger							reference			
Moderate hunger							−1.2 (−5.3; 3.0)			0.6
Severe hunger							22.1 (9.8; 34.4)			<0.001
Undernourished mother at delivery	5.0 (1.2; 8.8)	0.01								
	**Glutamic Acid (µg/mL)**	***p*** **Value**	**Glutamine (µg/mL)**	***p*** **Value**	**Glycine (µg/mL)**	***p*** **Value**	**Histidine (µg/mL)**	***p*** **Value**	**Leucine (µg/mL)**	***p*** **Value**
Meat, poultry, and fish					−6.6 (−12.5; −0.7)	0.03				
Red palm oil	−69.9 (−136.5; −3.4)	0.04			−5.0 (−9.3; −0.6)	0.03			25.7 (17.2; 34.1)	<0.001
Other oils and fats					2.2 (0.5; 3.8)	0.01				
Fish	−19.9 (−38.1; −1.8)	0.03								
HHS: Little to no hunger							reference			
Moderate hunger							−1.4 (−2.7; −0.1)	0.03		
Severe hunger							−0.9 (−4.8; 3.0)	0.7		
Undernourished mother at delivery	30.1 (10.3; 50.0)	0.003								
Milk-type group I	20.6 (−2.1; 43.2)	0.08	0.5 (−20.8; 21.7)	0.9						
Milk-type group II	−25.4 (−51.2; 0.3)	0.05	−24.8 (−48. 1; −1.5)	0.04						
Milk-type group III	reference		reference							
Milk-type group IV	2.0 (−30.8; 34.9)	0.90	−17.8 (−47.5; 11.9)	0.2						

Multilevel models with an intercept at the mother’s level were adjusted on the infant’s age. Adjusted beta-coefficients of the different variables are given with their 95% CI in parentheses. HFIAS: Household food insecurity access scale. HHS: Household hunger scale index.

**Table 10:** Multilevel models of the dietary determinants of human milk free amino acids during the first 6 months of life (2).

	Lysine (µg/mL)	*p* Value	Phenylalanine (µg/mL)	*p* Value	Serine (µg/mL)	*p* Value	Taurine (µg/mL)	*p* Value	Threonine (µg/mL)	*p* Value
Grains and white tubers									3.8 (1.6; 6.0)	0.001
Pulses: beans, peas, and lentils	3.9 (0.8; 7.0)	0.01							7.5 (1.9; 13.1)	0.01
Dark green, leafy vegetables							9.6 (2.7; 16.6)	0.01		
Red palm oil	6.1 (2.7; 9.5)	0.001	3.3 (1.8; 4.8)	<0.001						
HHS	−0.7 (−1.3; −0.0)	0.04								
Little to no hunger							reference		reference	
Moderate hunger							−7.9 (−15.1; −0.6)	0.03	−4.9 (−8.0; −1.7)	0.002
Severe hunger							−0.3 (−22.5; 21.9)	0.9	−6.1 (−15.5; 3.3)	0.2
Undernourished mother	−2.2 (−4.3; −0.2)	0.03								
Milk-type group I					−1.0 (−3.7; 1.6)	0.5				
Milk-type group II					−3.9 (−6.9; −1.0)	0.01				
Milk-type group III					reference					
Milk-type group IV					−3.1 (−6.8; 0.7)	0.1				
	**Tryptophan (µg/mL)**	***p*** **Value**	**Tyrosine (µg/mL)**	***p*** **Value**	**Valine (µg/mL)**		***p*** **Value**	**Sum of Free Amino Acids (µg/mL)**		***p*** **Value**
Grains, white roots, and tubers								53.2 (10.3; 96.2)		0.02
Pulses: beans, peas, and lentils								116.6 (7.2; 226.0)		0.04
Red palm oil	7.2 (5.9; 8.4)	<0.001								
HFIAS	−0.0 (−0.1; −0.0)	0.04								
HHS: Little to no hunger			reference		reference			reference		
Moderate hunger			−1.8 (−3.4; −0.3)	0.02	−3.0 (−5.6; −0.3)		0.03	−62.8 (−122.9; −2.8)		0.04
Severe hunger			−1.5 (−6.3; 3.2)	0.5	2.6 (−7.6; 12.7)		0.6	32.0 (−147.5; 211.6)		0.7
Milk-type group I			−1.6 (−2.9; −0.3)	0.02				10.4 (−35.3; 56.1)		0.7
Milk-type group II			−1.5 (−3.0 −0.0)	0.046				−83.9 (−136.4; −31.4)		0.002
Milk-type group III			reference					reference		
Milk-type group IV			−2.3 (−4.1; −0.5)	0.01				−21.5 (−85.5; 42.4)		0.5

Multilevel models with an intercept at the mother’s level were adjusted on the infant’s age. Adjusted beta-coefficients of the different variables are given with their 95% CI in parentheses. HFIAS: Household food insecurity access scale. HHS: Household hunger scale index.

## Data Availability

Concrete data are available upon request to the authors.

## Supplementary Materials

The following supporting information can be downloaded at: https://www.mdpi.com/article/10.3390/nu14194015/s1; Table S1: Questionnaires: 24-h recall and food consumption questionnaire; Table S2: Maternal diet and food security indexes during follow-up; Figure S1: Distribution of maternal diet during follow-up.
