# Supplementary material for: Food Insecurity and Maternal Diet Influence Human Milk Composition between the Infant’s Birth and 6 Months after Birth in Central-Africa
Source: Nutrients. 2022 Sep 27;14(19):4015. doi: 10.3390/nu14194015 (PMC9573613; doi:10.3390/nu14194015)
Supplement: Supplementary file 1 [file nutrients-14-04015-s001.zip › nutrients-1887566-supplementary-5.pdf]

Table S1- Questionnaires : 24-hour recall and food consumption questionnaire

24-HOUR RECALL (Mother)

Date of recall : /____/____/ 20__ Previous day : 1.Week day 2.Weekend day or public holiday /__/ Interviewer number: /__/

Food consumption : Yesterday, what did you eat ?

1) Meal : ________________________________ /__/__/__/ Sauce : ___________________ /__/__/__/
Composition : ____
____ At which moment of the day did you consume this dish? 1. Morning 2. Lunch 3. Evening 4. Afternoon 5. Mid-morning 6. Other. Please specify?____________
Where was this dish prepared? 1.At home 2. In the street 3. At the eatery 4.At the restaurant 5. Other. Please specify?_______________
When was this dish prepared ? 1. On the same day 2. The previous day 3. The previous week 4. Does not remember

2) Meal : ________________________________ /__/__/__/ Sauce : ___________________ /__/__/__/
Composition : ____
____ At which moment of the day did you consume this dish? 1.Morning 2.Lunch 3.Evening 4. Afternoon 5. Mid-morning 6. Other. Please specify?____________
Where was this dish prepared? 1.At home 2. In the street 3. At the eatery 4.At the restaurant 5. Other. Please specify?_______________
When was this dish prepared ? 1. On the same day 2. The previous day 3. The previous week 4. Does not remember

3) Meal : ________________________________ /__/__/__/ Sauce : ___________________ /__/__/__/
Composition : ____
____ At which moment of the day did you consume this dish? 1.Morning 2.Lunch 3.Evening 4. Afternoon 5. Mid-morning 6. Other. Please specify?____________
Where was this dish prepared? 1.At home 2. In the street 3. At the eatery 4. At the restaurant 5. Other. Please specify?_______________
When was this dish prepared ? 1. On the same day 2. The previous day 3. The previous week 4. Does not remember

4) Meal : ________________________________ /__/__/__/ Sauce : ___________________ /__/__/__/
Composition : ____
____ At which moment of the day did you consume this dish? 1.Morning 2.Lunch 3.Evening 4. Afternoon 5. Mid-morning 6. Other. Please specify?____________
Where was this dish prepared? 1.At home 2. In the street 3. At the eatery 4.At the restaurant 5. Other. Please specify?_______________
When was this dish prepared ? 1. On the same day 2. The previous day 3. The previous week 4. Does not remember

Snacks consumption: Yesterday, which snacks did you eat?

5) _______________ /__/__/__/ (detail) :___________ Composition : _________________________

6) _______________ /__/__/__/ (detail) :___________ Composition : _________________________

7) _______________ /__/__/__/ (detail) :___________ Composition : _________________________

8) _______________ /__/__/__/ (detail) :___________ Composition: _________________________

Beverage consumption: Yesterday, which beverages did you drink?

9) _______________ /__/__/__/ (detail) :________________ Composition : _______________________

10) _______________ /__/__/__/ (detail) :________________ Composition : _______________________

11) _______________ /__/__/__/ (detail) :________________ Composition : _______________________

12) _______________ /__/__/__/ (detail) :________________ Composition : _______________________

Fruit consumption: Yesterday, which fruits did you consume?

13) _______________ 14) _______________

15) _______________ 16) _______________

17) _______________ 18) _______________

Was your food and beverage consumption yesterday "unusual" ? 1.yes 2.no /__/

If yes, was your food and beverage consumption : 1.Higher than usual (wedding, …) 2.Smaller than usual (travel-ling,…) /__/

HOUSEHOLD FOOD INSECURITY ACCESS SCALE	
For Each of the questions, consider what happened in the PAST 30 DAYS	***Please answer if it happened:***<br>***- NEVER,***<br>***- RARELY (once or twice in the past 30 days),***<br>***- SOMETIMES (three to ten times in the past 30 days),***<br>***- OFTEN (almost every day, more than ten times in the past 30 days).***
In the past 30 days, did you WORRY that your household would not have enough food?	
In the past 30 days, were you or any household member not able to eat the KINDS OF FOODS YOU PREFERRED because of a lack of resources??	
In the past 30 days, did you or any household member have to eat a LIMITED VARIETY OF FOODS due to a lack of resources?	
In the past 30 days, did you or any household member have to eat SOME FOODS THAT YOU REALLY DID NOT WANT TO EAT because of a lack of resources to obtain other types of food??	
In the past 30 days, did you or any household member have to eat a SMALLER MEAL than you felt you needed because there was not enough food??	
In the past 30 days, did you or any household member have to eat FEWER meals in a day because there was not enough food??	
In the past 30 days, was there ever NO FOOD TO EAT of any kind in your household because of lack of resources to get food?	
In the past 30 days, did you or any household member go to sleep at night HUNGRY because there was not enough food?	
In the past 30 days, did you or any household member go A WHOLE DAY AND NIGHT WITHOUT EATING anything because there was not enough food??	
**FOOD CONSUMPTION OF THE MOTHER**	
Was your food and beverage consumption yesterday unusual compared to the rest of the week ? (Celebration, ceremony, or on the opposite a missed meal ?)	
Yesterday, was your food and beverage consumption ger or smaller than usual?	
Larger	
The same	

Smaller

Watch out ! Please write down all food and beverages consumed on the questionnaire provided for this purpose. Remember to describe the meals!

Did you eat yesterday morning?

Did you eat yesterday at lunch?

Did you eat yesterday evening?

Did you eat anything else during the day in the morning or in the afternoon outside of these meals?

Number of meals consumed during the previous day?

Number of meals during any normal given day?

ALL MEALS consumed by the MOTHER on the PREVIOUS DAY

Cereals	Millet, sorghum, fonio, rice, corn, wheat… including bread, pasta, couscous, dough-nuts, flour....
Orange-flesh sweet potato	
Roots and tubers	Cassava, yam, white/pale yellow fleshed sweet potato, taro root, fabirama (pesa), po-tatoes
Pulses	Beans, Bambara groundnut, sweet pea, lentil, dried pulses, soybean, etc.
Peanut, groundnut	To eat, or in a sauce
Sesame seed	Ground, as seeds in a sauce, on pancakes
Nuts and seeds	Cashew, wild nuts, other nuts and seeds rich in oil (EXCPET for cola nuts to chew)
Dark green leady vegetables	Sorrel and hibiscus leaves, spinach, sweet potato leaves, moringa and peanut leaves, baobab greens, onion leaves, bean leaves, etc.
Dark orange- and red-flesh vegetables	Pumpkin, carrots, red pepper
Tomato concentrate	
Other vegetables	Tomato, okra, zucchini, eggplant, onion, cabbage, cucumber, salad, etc.
Vitamin A rich fruits	Mango, red or dark-orange fleshed papaya, dark (orange fleshed melon, African locust bean (including juices)
Other fruits	Banana, pineapple, tamarind, monkey's bread, grapes, watermelon, wild fruits... (in-cluding fresh fruit juice).
Liver	Beef, veal, sheep, poultry…
Meat and other offals	In sticks, stews (beef, sheep, chicken and poultry, goat, pork, dog, etc) including offals apart from liver
Meat, poultry, offals as condiments	In a sauce or in a soup
Insects, small rodents and other small animals	
Fish powder, dried, salted or smoked	Or ground dried fish used as condiment in a sauce
Small dried fishes	
Other fishes and seafood	Fresh, or preserved
Eggs	From chiken, guinea fowl
Milk and milk products	Goat, cow, camel milk,… powdered, concentrated, fermented
Red palm oil	Or red palm fruit/pulp
Other oils and fats	Vegetable oils, butter, shea butter, margarine and mayonnaise, fried foods
Cakes and pastries	
Tea, coffee	Please detail with or without milk and sugar
Sugar, honey, jam, candies	
Sweet beverages	Fanta and other soft drinks, modji, ginger juice, tamarind juice, hibiscus juice, mon-key's bread juice, etc.

Alcoholic beverages	Beer, millet beer, palm wine, spirits,...
Condiments	Salt, potash, pepper, chili, ginger, "datou", African locust bean powder, stock cube, tomato powder, tomato concentrate, etc.
Number of snacks and collation consumed by the mother on the previous day	

Table S2. Maternal diet and food security indexes during follow-up

Maternal diet (food-consumption questionnaire)	first week	non-undernourished women	undernourished women	*P* value of the difference	4 weeks	non-undernourished women	undernourished women	*P* value of the difference	11 weeks	non-undernourished women	undernourished women	*P* value of the difference
Grains, white roots and tubers	41/44 (93.2%)	23/26 (88.5%)	14/14 (100.0%)	0.5	40/40 (100.0%)	26/26 (100.0%)	12/12 (100.0%)	0.9	38/38 (100.0%)	25/25 (100.0%)	11/11 (100.0%)	0.9
Pulses: beans, peas and lentils	5/43 (11.6%)	4/25 (16.0%)	0/14 (0.0%)	0.3	4/39 (10.3%)	3/26 (11.5%)	0/11 (0.0%)	0.5	3/38 (7.9%)	2/25 (8.0%)	1/11 (9.1%)	0.9
Nuts and seeds	24/43 (55.8%)	13/25 (52.0%)	10/14 (71.4%)	0.3	26/39 (66. 7%)	16/25 (64.0%)	8/12 (66.7%)	0.9	29/38 (92.3%)	19/25 (76.0%)	8/11 (72.7%)	0.9
Milk and milk products	14/42 (33.3%)	7/25 (28.0%)	5/13 (38.5%)	0.7	17/41 (42.5%)	15/26 (57.7%)	1/12 (8.3%)	**0.01**	14/38 (36.8%)	10/25 (40.0%)	2/11 (18.2%)	0.3
Meat, poultry, and fish	32/41 (78.1%)	18/24 (75.0%)	10/13 (76.9%)	0.9	36/40 (90.0%)	25/26 (96.2%)	9/12 (75.0%)	0.1	14/38 (76.3%)	18/25 (72.0%)	9/11 (81.8%)	0.7
Eggs	2/42 (4.8%)	0/25 (00.0%)	1/13 (7.7%)	0.3	0	0/26 (0.0%)	0/12 (0.0%)	0.9	0	0/25 (0.0%)	0/11 (0.0%)	0.9
Dark green leafy vegetables	16/43 (37.2%)	6/25 (24.0%)	9/14 (64.3%)	**0.02**	18/40 (45.0%)	9/26 (34.6%)	7/12 (58.3%)	0.3	23/38 (60.5%)	14/25 (56.0%)	8/11 (72.3%)	0.5
Other vitamin A rich fruits and vegetables	15/42 (35.7%)	10/25 (40.0%)	4/13 (30.8%)	0.7	13/40 (32.5%)	7/26 (26.9%)	5/12 (41.7%)	0.5	11/38 (29.0%)	8/25 (32.0%)	2/11 (18.2%)	0.7
Other vegetables	31/43 (72.1%)	18/25 (72.0%)	10/14 (71.4%)	0.9	37/40 (92.5%)	24/26 (92.3%)	11/12 (91.7%)	0.9	34/38 (89.5%)	21/25 (84.0%)	11/11 (100.0%)	0.3
Other fruits	4/42 (9.5%)	2/25 (8.0%)	2/13 (15.4%)	0.6	7/40 (17.5%)	4/26 (15.4%)	3/12 (25.0%)	0.7	3/38 (7.9%)	1/25 (4.0%)	1/11 (9.1%)	0.5
Insects, small rodents and other small animals	4/42 (9.5%)	2/25 (8.0%)	2/13 (15.4%)	0.6	1/40 (2.5%)	1/26 (3.9%)	0/12 (0.0%)	0.9	7/38 (18.4%)	6/25 (24.0%)	1/11 (9.1%)	0.4
Red palm oil	10/42 (23.8%)	6/25 (24.0%)	4/13 (30.8%)	0.7	11/40 (27.5%)	5/26 (19.2%)	5/12 (41.7%)	0.2	8/38 (21.1%)	6/25 (24.0%)	1/11 (9.1%)	0.4
Other oils and fats	36/42 (85.7%)	20/25 (80.0%)	12/13 (92.3%)	0.6	34/40 (85.00%)	23/26 (88.5%)	9/12 (75.0%)	0.4	33/38 (86.8%)	22/25 (88.0%)	10/11 (90.9%)	0.9
Condiments and seasonings	37/43 (86.1%)	22/25 (88.0%)	11/14 (78.6%)	0.6	31/40 (77.5%)	21/26 (80.8%)	8/12 (66.7%)	0.4	29/38 (76.3%)	19/25 (76.0%)	8/11 (72.7%)	0.9
Other beverages and foods	25/42 (59.5%)	10/25 (40.0%)	11/13 (84.6%)	**0.02**	23/40 (57.5%)	10/26 (38.5%)	11/12 (91.7%)	**0.004**	20/38 (52.6%)	9/25 (36.0%)	9/11 (81.8%)	**0.03**
Sweet foods	35/43 (81.4%)	18/25 (72.0%)	14/14 (100.0%)	**0.04**	35/40 (87.5%)	22/26 (84.6%)	11/12 (91.7%)	0.9	34/38 (89.5%)	21/25 (84.0%)	11/11 (100.0%)	0.3
Sweet beverages	1/43 (2.3%)	1/25 (4.0%)	0/14 (0.0%)	0.9	1/40 (2.5%)	1/26 (3.9%)	0/12 (0.0%)	0.9	0	0/25 (0.0%)	0/11 (0.0%)	0.9
Woman's dietary diversity score (WDDS)	4.00(3.0 ; 6.0)	4.00 (2.0 ; 5.0)	5.0 (4.0 ; 6.0)	0.2	5.0 (4.0 ; 6.0)	5.0 (4.0 ; 6.0)	5.0 (3.5 ; 6.0)	0.8	5.0 (4.0 ; 6.0)	5.00 (4.0 ; 6.0)	5.00 (4.0 ; 6.0)	0.9
WDDS categories				0.3				0.9				0.7
Low diverse diet (WDD < 5)	24/42 (57.1%)	16/26 (61.5%)	6/14 (42.9%)		16/40 (40.0%)	11/26 (42.3%)	5/12 (41.7%)		15/38 (39.5%)	11/25 (44.0%)	4/11 (36.4%)	

Diverse diet (WDD >= 5)	18/42 (42.9%)	10/26 (38.5%)	8/14 (57.1%)		24/40 (60.0%)	15/26 (57.7%)	7/12 (58.3%)		23/38 (60.5%)	14/25 (56.0%)	7/11 (63.6%)	
**Maternal diet (24-h recall)**												
Grains, white roots and tubers	32/32 (100.0%)	19/19 (100.0%)	12/12 (100.0%)	0.9	29/29 (100.0%)	20/20 (100.0%)	9/9 (100.0%)	0.9	31/31 (100.0%)	20/20 (100.0%)	11/11 (100.0%)	0.9
Pulses: beans, peas and lentils	2/32 (6.3%)	2/19 (10.5%)	0/12 (0.0%)	0.5	1/29 (3.5%)	1/20 (5.0%)	0/9 (0.0%)	0.9	1/31 (3.2%)	0/20 (0.0%)	1/11 (9.1%)	0.4
Nuts and seeds	15/32 (46.9%)	6/19 (31.6%)	8/12 (66.7%)	0.1	22/29 (75.9%)	15/20 (75.0%)	7/9 (77.8%)	0.9	20/31 (64.5%)	12/20 (60.0%)	8/11 (72.7%)	0.7
Milk and milk products	11/32 (34.4%)	9/19 (47.4%)	1/12 (8.3%)	**0.046**	12/29 (41.4%)	11/20 (55.0%)	1/9 (11.1%)	**0.04**	9/31 (29.0%)	8/20 (40.0%)	1/11 (9.1%)	0.1
Meat, poultry, and fish	27/32 (84.4%)	17/19 (89.5%)	9/12 (75.0%)	0.4	26/29 (89.7%)	20/20 (100.0%)	6/9 (66.7%)	**0.02**	26/31 (83.9%)	16/20 (80.0%)	10/11 (90.9%)	0.6
Eggs	0/32 (0.0%)	0/19 (0.0%)	0/12 (0.0%)	0.9	0/29 (0.0%)	0/20 (0.0%)	0/9 (0.0%)	0.9	0/31 (0.0%)	0/20 (0.0%)	0/11 (0.0%)	0.9
Dark green leafy vegetables	16/32 (50.0%)	6/19 (31.6%)	10/12 (83.3%)	**0.009**	19/29 (65.5%)	11/20 (55.00%)	8/9 (88.9%)	0.1	20/31 (64.5%)	13/20 (65.0%)	7/11 (63.6%)	0.9
Other vitamin A rich fruits and vegetables	6/32 (18.8%)	4/19 (21.1%)	1/12 (8.3%)	0.6	1/29 (3.5%)	1/20 (5.00%)	0/9 (0.0%)	0.9	3/31 (9. 7%)	2/20 (10.0%)	1/11 (9.1%)	0.9
Other vegetables	30/32 (93.8%)	17/19 (89.5%)	12/12 (100.0%)	0.5	26/29 (89.7%)	19/20 (95.00%)	7/9 (77.8%)	0.2	28/31 (90.3%)	18/20 (90.0%)	10/11 (90.9%)	0.9
Other fruits	3/32 (9.4%)	1/19 (5.3%)	2/12 (16.7%)	0.5	3/29 (10.3%)	3/20 (15.00%)	0/9 (0.0%)	0.5	3/31 (9.7%)	1/20 (5.0%)	2/11 (18.2%)	0.3
Insects, small rodents and other small animals	3/32 (9.4%)	1/19 (5.3%)	2/12 (16.7%)	0.5	3/29 (10.3%)	3/20 (15.00%)	0/9 (0.0%)	0.5	5/31 (16.1%)	4/20 (20.0%)	1/11 (9.1%)	0.6
Red palm oil	10/32 (31.3%)	5/19 (26.3%)	4/12 (33.3%)	0.7	13/29 (44.8%)	5/20 (25.0%)	8/9 (88.9%)	**0.003**	7/31 (22.6%)	4/20 (20.0%)	3/11 (27.3%)	0.7
Other oils and fats	31/32 (96. 9%)	18/19 (94.7%)	12/12 (100.0%)	0. 9	29/29 (100.0%)	20/20 (100.0%)	9/9 (100.0%)	0.9	28/31 (90.3%)	18/20 (90.0%)	10/11 (90.9%)	0.9
Condiments and seasonings	31/32 (96.9%)	18/19 (94.7%)	12/12 (100.0%)	0.9	28/29 (96.6%)	20/20 (100.0%)	8/9 (88.9%)	0.3	29/31 (93.6%)	18/20 (90.0%)	11/11 (100.0%)	0.5
Other beverages and foods	1/32 (3.1%)	1/19 (5.3%)	0/12 (0.0%)	0.9	2/29 (6.9%)	1/20 (5.0%)	1/9 (11.1%)	0.5	2/31 (6.5%)	1/20 (5.0%)	1/11 (9.1%)	0.9
Sweet foods	13/32 (40.6%)	7/19 (36.8%)	6/12 (50.0%)	0.7	11/29 (37.9%)	6/20 (30.0%)	5/9 (55.6%)	0.2	12/31 (38.7%)	8/20 (40.0%)	4/11 (36.4%)	0.9
Sweet beverages	12/32 (37.5%)	6/19 (31.6%)	6/12 (50.0%)	0.5	13/29 (44.8%)	7/20 (35.0%)	6/9 (66.7%)	0.2	11/31 (35.5%)	6/20 (30.0%)	5/11 (45.5%)	0.5
Woman's dietary diversity score (WDDS)	5.0 (4.0 ; 5.0)	4.0 (4.0 ; 5.0)	5.0 (4.0 ; 5.0)		5.0 (4.0 ; 6.0)	5.0 (4.0 ; 6.0)	5.0 (3.0 ; 5.0)		5.0 (3.0 ; 5.0)	5.0 (3.5 ; 5.0)	5.00 (3.0 ; 6.0)	
WDDS categories												
Low diverse diet (WDD < 5)	14/32 (43.8%)	10/19 (52.6%)	4/12 (33.3%)		11/29 (37.9%)	7/20 (35.0%)	4/9 (44.4%)		12/31 (38.7%)	8/20 (40.0%)	4/11 (36.4%)	
Diverse diet (WDD >= 5)	18/32 (56.3%)	9/19 (47.4%)	8/12 (66.7%)		18/29 (62.1%)	13/20 (65.0%)	5/9 (55.6%)		19/31 (61.3%)	12/20 (60.0%)	7/11 (63.6%)	
**Food security during follow-up**												

Household Food Insecurity Access Scale (HFIAS)	9.0 (6.0 ; 12.0)	8.0 (5.0 ; 11.5)	11.0 (9.0 ; 13.0)	**0.049**	8.0 (6.0 ; 11.0)	8.0 (6.0 ; 10.0)	11.0 (8.0 ; 12.0)	0.1	9.0 (7.0 ; 14.0)	9.0 (7.0 ; 12.0)	11.0 (8.0 ; 16.0)	0.2
Categories of HFIAS				0.5				0.4				0.5
Food secure	1/48 (2.1%)	1/28 (3.6%)	0		0	0	0		0	0	0	
Mildly food insecure	0	0	0		1/41 (2.4%)	0	1/13 (7.7%)		2/40 (5.0%)	2/25 (8.0%)	0	
Moderately food insecure	22/48 (45.8%)	16/28 (57.1%)	6/15 (40.0%)		21/41 (51.2%)	14/26 (53.9%)	5/13 (38.5%)		18/40 (45.0%)	12/25 (48.0%)	5/13 (38.5%)	
Severely food insecure	25/48 (52.1%)	11/28 (39.3%)	9/15 (60.0%)		19/41 (46.3%)	12/26 (46.2%)	7/13 (53.9%)		20/40 (50.0%)	11/25 (44.0%)	8/13 (61.5%)	
Household hunger scale index (HHS)	0.0 (0.0 ; 1.0)	0.0 (0.0 ; 1.5)	0.0 (0.0 ; 2.0)	0.7	0.0 (0.0 ; 1.0)	0.0 (0.0 ; 1.0)	0.0 (0.0 ; 1.0)	0.5	0.0 (0.0 ; 1.0)	0.0 (0.0 ; 1.0)	1.0 (0.0 ; 1.0)	0.1
Categores of HHS				0.9				0.6				0.6
Little to no hunger in the household	35/46 (76.1%)	21/28 (75.0%)	11/15 (73.3%)		36/41 (87.8%)	22/26 (84.6%)	12/13 (92.3%)		36/40 (90.0%)	23/25 (92.0%)	11/13 (84.6%)	
Moderate hunger in the household	10/46 (21.7%)	6/28 (21.4%)	4/15 (26.7%)		5/41 (12.2%)	4/26 (15.4%)	1/13 (7.7%)		4/40 (10.0%)	2/25 (8.0%)	2/13 (15.4%)	
Severe hunger in the household	1/46 (2.2%)	1/28 (3.6%)	0		0	0	0		0	0	0	

Table S2. Maternal diet and food security indexes during follow-up (continuation)

**Maternal diet (food-consumption questionnaire)**	**18 weeks**	**non-undernourished women**	**undernourished women**	***P* value of the difference**	**25 weeks**	**non-undernourished women**	**undernourished women**	***P* value of the difference**	***P* value of the difference between non-undernourished and undernourished during the entire follow-up***	***P* value of the differences in food consumption during the entire follow-up***
Grains, white roots and tubers	28/29 (96.6%)	14/15 (93.3%)	12/12 (100.0%)	0.9	34/34 (100.0%)	20/20 (100.0%)	12/12 (100.0%)	0.9	0.9	0.2
Pulses: beans, peas and lentils	2/29 (6.9%)	0/15 (0.0%)	2/12 (16.7%)	0.2	2/34 (5.9%)	1/20 (5.0%)	1/12 (8.3%)	0.9	0.7	0.4
Nuts and seeds	25/29 (86.2%)	13/15 (86.7%)	10/12 (83.3%)	0.9	26/34 (76.5%)	14/20 (70.0%)	10/12 (83.3%)	0.7	0.3	**0.01**

Milk and milk products	9/29 (31.0%)	5/15 (33.3%)	3/12 (25.0%)	0.7	15/34 (44.1%)	10/20 (50.0%)	4/12 (33.3%)	0.5	0.1	0.5
Meat, poultry, and fish	20/29 (69.0%)	9/15 (60.0%)	9/12 (75.0%)	0.7	27/34 (79.4%)	16/20 (80.0%)	9/12 (75.0%)	0.9	0.8	0.5
Eggs	2/29 (6.9%)	0/15 (0.0%)	2/12 (16.7%)	0.2	1/34 (2.9%)	1/20 (5.0%)	0/12 (0.0%)	0.9	0.1	0.8
Dark green leafy vegetables	20/29 (69.0%)	9/15 (60.0%)	9/12 (75.0%)	0.7	26/34 (76.5%)	14/20 (70.0%)	10/12 (83.3%)	0.7	**0.01**	**<0.001**
Other vitamin A rich fruits and vegetables	8/29 (27.6%)	1/15 (6.7%)	7/12 (58.3%)	**0.008**	15/34 (44.1%)	7/20 (35.0%)	8/12 (66.7%)	0.1	0.1	0.6
Other vegetables	27/29 (93.1%)	14/15 (93.3%)	11/12 (91.7%)	0.9	33/34 (97.1%)	20/20 (100.0%)	11/12 (91.7%)	0.4	0.8	**0.005**
Other fruits	3/29 (10.34%)	1/15 (6.7%)	2/12 (16.7%)	0.6	0	0/20 (0.0%)	0/12 (0.0%)	0.9	0.2	0.1
Insects, small rodents and other small animals	6/29 (20.69%)	5/15 (33.3%)	1/12 (8.3%)	0.2	3/34 (8.8%)	3/20 (15.0%)	0/12 (0.0%)	0.3	0.1	0.3
Red palm oil	7/29 (24.14%)	1/15 (6.7%)	6/12 (50.0%)	**0.02**	9/34 (26.5%)	4/20 (20.0%)	5/12 (41.7%)	0.2	0.1	0.9
Other oils and fats	26/29 (89.7%)	14/15 (93.3%)	10/12 (83.3%)	0.6	33/34 (97.1%)	20/20 (100.0%)	11/12 (91.7%)	0.4	0.6	0.1
Condiments and seasonings	24/29 (82.8%)	11/15 (73.3%)	11/12 (91.7%)	0.3	27/34 (79.4%)	17/20 (85.0%)	8/12 (66.7%)	0.4	0.4	0.7
Other beverages and foods	16/29 (55.2%)	4/15 (26.7%)	11/12 (91.7%)	**0.001**	17/34 (50.0%)	6/20 (30.0%)	10/12 (83.3%)	**0.009**	**<0.001**	0.3
Sweet foods	24/29 (82.8%)	12/15 (80.00%)	10/12 (83.3%)	0.9	31/34 (91.2%)	18/20 (90.0%)	11/12 (91.7%)	0.9	0.048	0.3
Sweet beverages	0	0/15 (0.0%)	0/12 (0.0%)	0.9	0	0/20 (0.0%)	0/12 (0.0%)	0.9	0.9	0.5
Woman's dietary diversity score (WDDS)	5.0 (4.0 ; 6.0)	4.0 (4.0 ; 5.0)	6.0 (4.0 ; 7.0)	0.1	5.0 (5.0 ; 6.0)	5.0 (4.0 ; 6.0)	5.00 (5.0 ; 6.0)	0.5	0.1	**0.002**
WDDS categories				0.3				0.2	0.1	**0.01**
Low diverse diet (WDD < 5)	13/29 (44.8%)	9/15 (60.0%)	4/12 (33.3%)		8/34 (23.5%)	7/20 (35.0%)	1/12 (8.3%)			
Diverse diet (WDD >= 5)	16/29 (55.2%)	6/15 (40.0%)	8/12 (66.7%)		26/34 (76.5%)	13/20 (65.0%)	11/12 (91.7%)			
**Maternal diet (24-h recall)**										
Grains, white roots and tubers	23/23 (100.0%)	11/11 (100.0%)	11/11 (100.0%)	0.9	22/22 (100.0%)	14/14 (100.0%)	7/7 (100.0%)	0.9	0.9	0.9

Pulses: beans, peas and lentils	2/23 (8.7%)	0/11 (0.0%)	2/11 (18.2%)	0.5	1/22 (4.6%)	1/14 (7.1%)	0/7 (0.0%)	0.9	0.7	0.4
Nuts and seeds	16/23 (69.6%)	8/11 (72.7%)	7/11 (63.6%)	0.9	15/22 (68.2%)	11/14 (78.6%)	4/7 (57.1%)	0.4	0.3	0.1
Milk and milk products	7/23 (30.4%)	4/11 (36.4%)	2/11 (18.2%)	0.6	5/22 (22.7%)	4/14 (28.6%)	1/7 (14.3%)	0.6	**0.004**	0.3
Meat, poultry, and fish	15/23 (65.2%)	6/11 (54.6%)	8/11 (72.7%)	0.7	20/22 (90.9%)	13/14 (92.9%)	6/7 (85.7%)	0.9	0.3	0.9
Eggs	1/23 (4.4%)	0/11 (0.0%)	1/11 (9.1%)	0.9	0/22 (0.0%)	0/14 (0.0%)	0/7 (0.0%)	0.9	0.7	0.7
Dark green leafy vegetables	16/23 (69.6%)	7/11 (63.6%)	8/11 (72.7%)	0.9	17/22 (77.3%)	11/14 (78.6%)	5/7 (71.4%)	0.9	**0.02**	**0.03**
Other vitamin A rich fruits and vegetables	3/23 (13.0%)	1/11 (9.1%)	2/11 (18.2%)	0.9	2/22 (9.1%)	0/14 (0.0%)	2/7 (28.6%)	0.1	0.9	0.8
Other vegetables	23/23 (100.0%)	11/11 (100.0%)	11/11 (100.0%)	0.9	20/22 (90.9%)	13/14 (92.9%)	6/7 (85.7%)	0.9	0.7	0.7
Other fruits	3/23 (13.0%)	2/11 (18.2%)	0/11 (0.0%)	0.5	2/22 (9.1%)	2/14 (14.3%)	0/7 (0.0%)	0.5	0.3	0.9
Insects, small rodents and other small animals	5/23 (21.7%)	5/11 (45.5%)	0/11 (0.0%)	**0.04**	2/22 (9.1%)	2/14 (14.3%)	0/7 (0.0%)	0.5	0.1	0.6
Red palm oil	7/23 (30.4%)	3/11 (27.3%)	4/11 (36.4%)	0.9	3/22 (13.6%)	1/14 (7.1%)	2/7 (28.6%)	0.2	**0.01**	0.7
Other oils and fats	23/23 (100.0%)	11/11 (100.0%)	11/11 (100.0%)	0.9	21/22 (95.5%)	14/14 (100.0%)	6/7 (85.7%)	0.3	0.8	0.2
Condiments and seasonings	22/23 (95.7%)	11/11 (100.0%)	10/11 (90.9%)	0.9	22/22 (100.0%)	14/14 (100.0%)	7/7 (100.0%)	0.9	0.7	0.4
Other beverages and foods	2/23 (8.7%)	0/11 (0.0%)	2/11 (18.2%)	0.5	0/22 (0.0%)	0/14 (0.0%)	0/7 (0.0%)	0.9	0.8	0. 2
Sweet foods	11/23 (47.8%)	4/11 (36.4%)	6/11 (54.6%)	0.7	8/22 (36.4%)	4/14 (28.6%)	4/7 (57.1%)	0.3	0.9	0.6
Sweet beverages	12/23 (52.2%)	4/11 (36.4%)	7/11 (63.6%)	0.4	7/22 (31.8%)	4/14 (28.6%)	3/7 (42.9%)	0.6	**0.04**	0.7
Woman's dietary diversity score (WDDS)	5.0 (4.0 ; 6.0)	5.0 (3.0 ; 6.0)	4.0 (4.0 ; 6.0)		5.0 (3.0 ; 6.0)	5.0 (4.0 ; 6.0)	4.0 (3.0 ; 6.0)		0.6	0.2
WDDS categories									0.6	0.6
Low diverse diet (WDD < 5)	10/23 (43.5%)	4/11 (36.4%)	6/11 (54.6%)		9/22 (40.9%)	4/14 (28.6%)	4/7 (57.1%)			
Diverse diet (WDD >= 5)	13/23 (56.5%)	7/11 (63.6%)	5/11 (45.5%)		13/22 (59.1%)	10/14 (71.4%)	3/7 (42.9%)			
**Food security during follow-up**										

Household Food Insecurity Access Scale (HFIAS)	10.0 (6.0 ; 12.0)	8.0 (6.0 ; 12.0)	10.0 (7.0 ; 14.5)	0.4	8.0 (5.0 ; 12.0)	8.00 (5.0 ; 13.0)	8.50 (4.0 ; 11.5)	0.6	0.1	0.2
Categories of HFIAS				0.8				0.6	0.9	0.9
Food secure	0	0	0		1/33 (3.0%)	0	1/12 (8.3%)			
Mildly food insecure	1/28 (3.6%)	0	1/12 (8.3%)		1/33 (3.0%)	1/19 (5.3%)	0			
Moderately food insecure	15/28 (53.6%)	8/14 (57.1%)	6/12 (50.0%)		15/33 (45.5%)	8/19 (42.1%)	6/12 (50.0%)			
Severely food insecure	12/28 (42.9%)	6/14 (42.9%)	5/12 (41.7%)		16/33 (48.5%)	10/19 (52.6%)	5/12 (41.7%)			
Household hunger scale index (HHS)	0.0 (0.0 ; 1.0)	0.00 (0.0 ; 1.0)	0.0 (0.0 ; 1.0)	0.9	0.0 (0.0 ; 1.0)	0.0 (0.0 ; 1.0)	0.0 (0.0 ; 0.5)	0.3	0.6	0.2
Categores of HHS				0.9				0.7	0.7	0.1
Little to no hunger in the household	27/29 (93.1%)	14/15 (93.3%)	11/12 (91.7%)		28/33 (84.9%)	17/19 (89.5%)	10/12 (83.3%)			
Moderate hunger in the household	2/29 (6.9%)	1/15 (6.7%)	1/12 (8.3%)		4/33 (12.1%)	1/19 (5.3%)	2/12 (16.7%)			
Severe hunger in the household	0	0	0		1/33 (3.0%)	1/19 (5.3%)	0			

Figure S1. Distribution of maternal diet during follow-up

One week after delivery

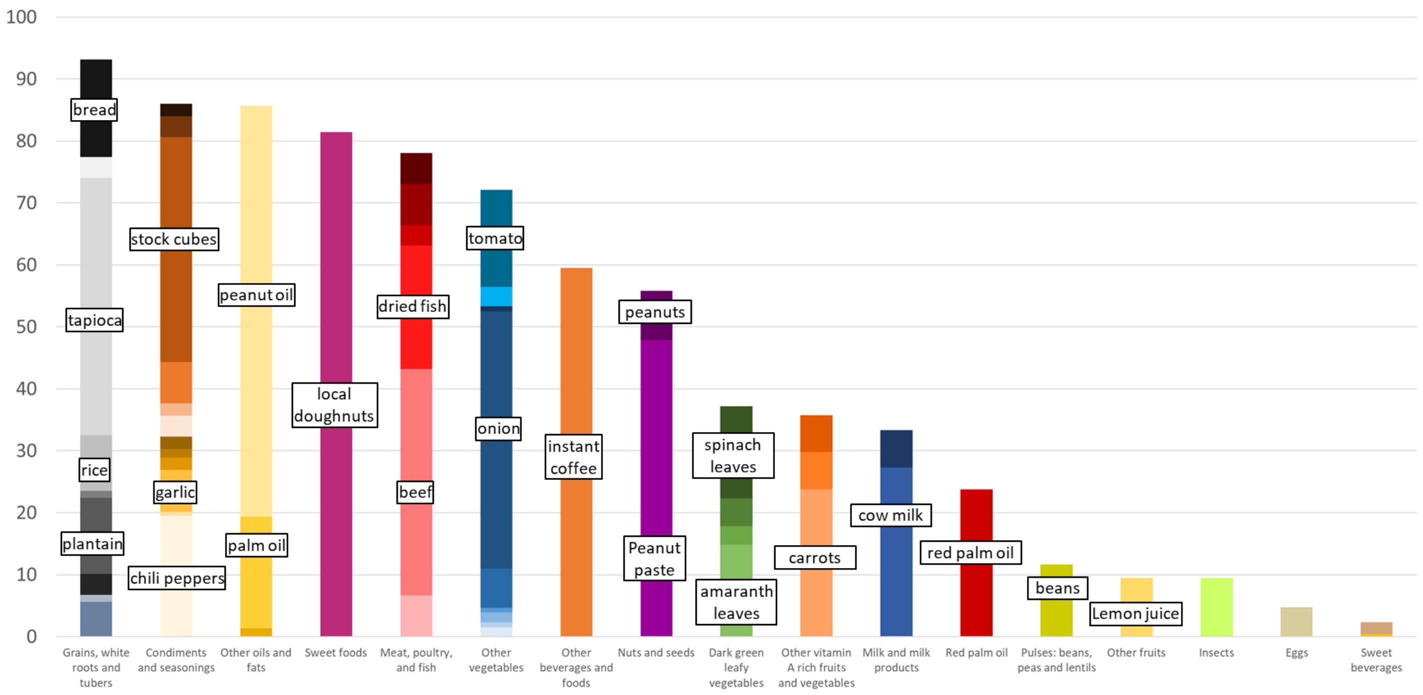

4 weeks

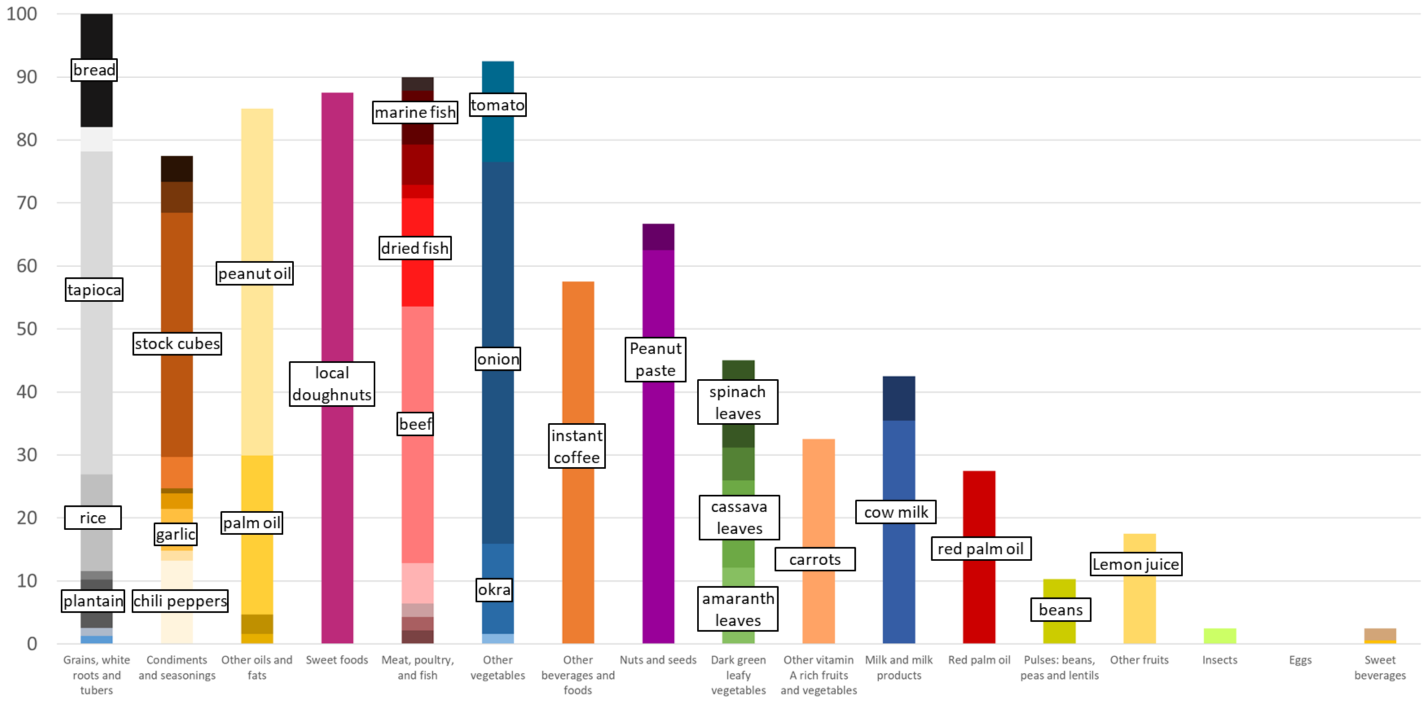

11 weeks

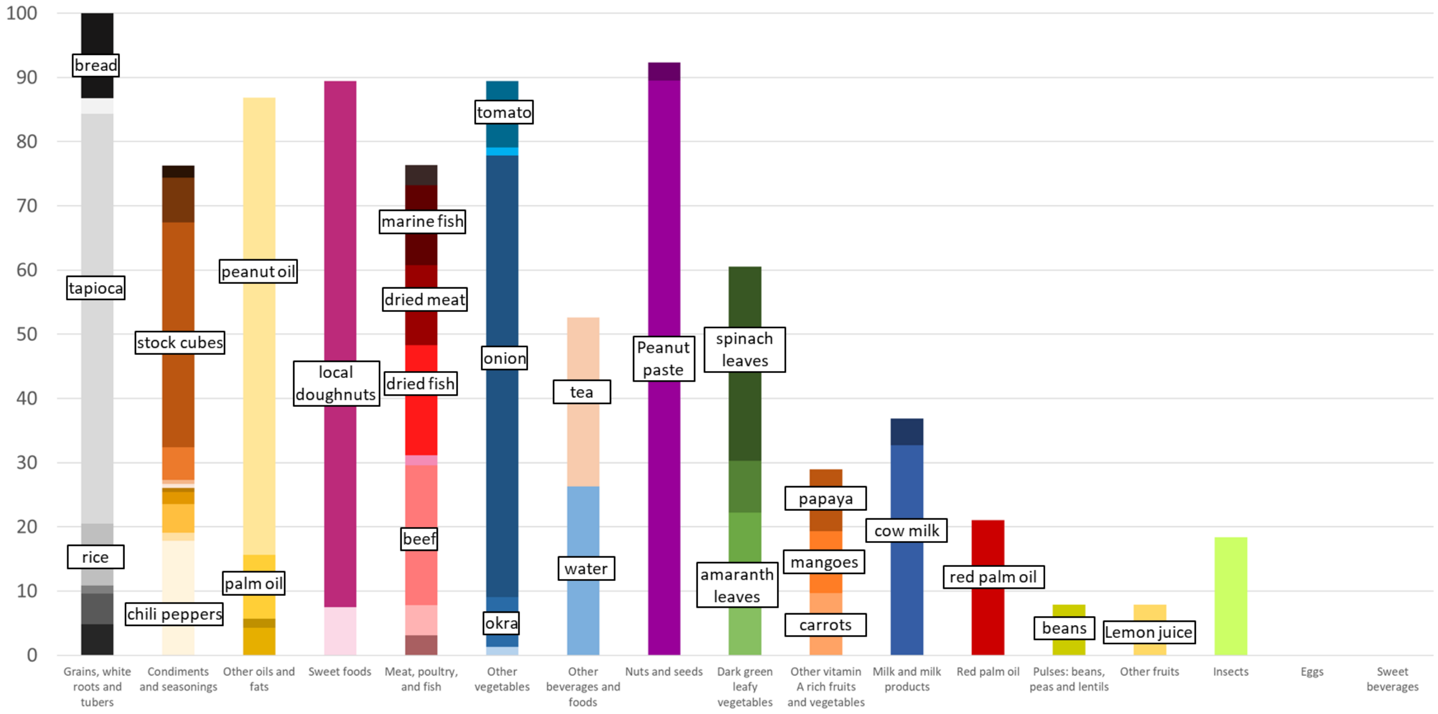

18 weeks

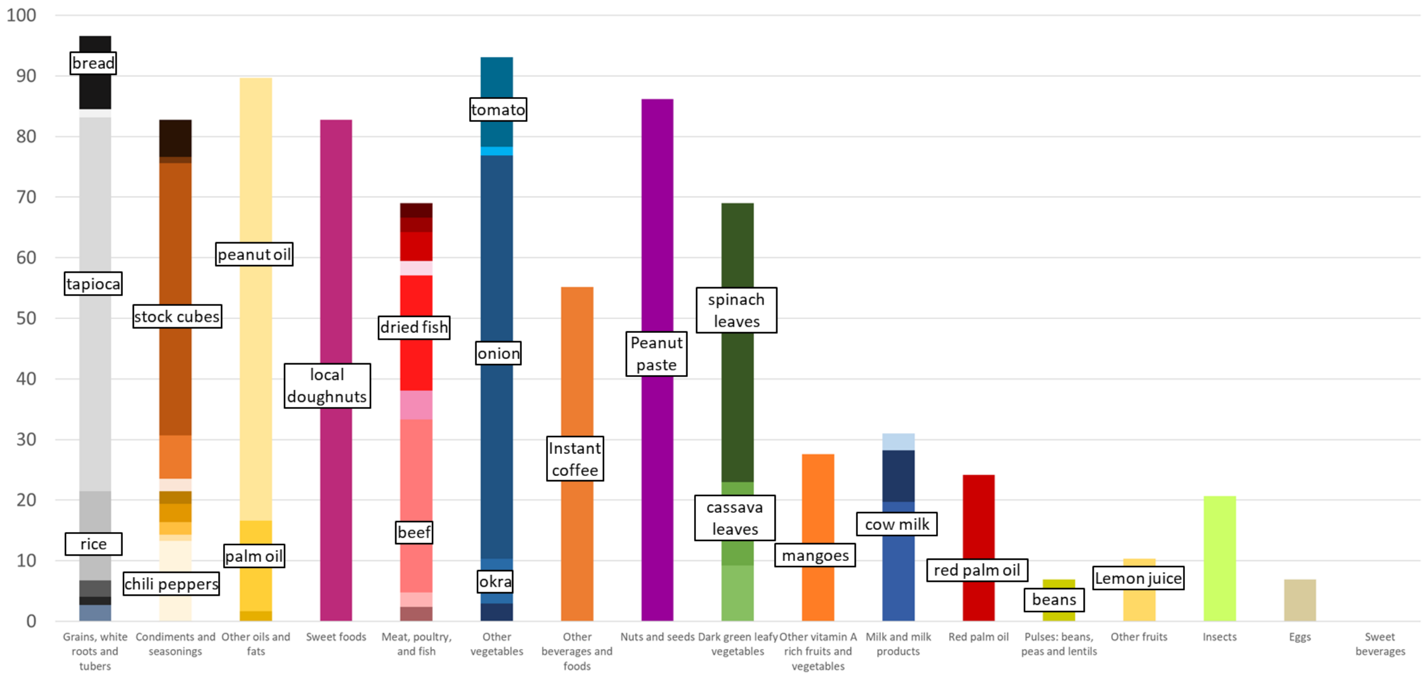

25 weeks

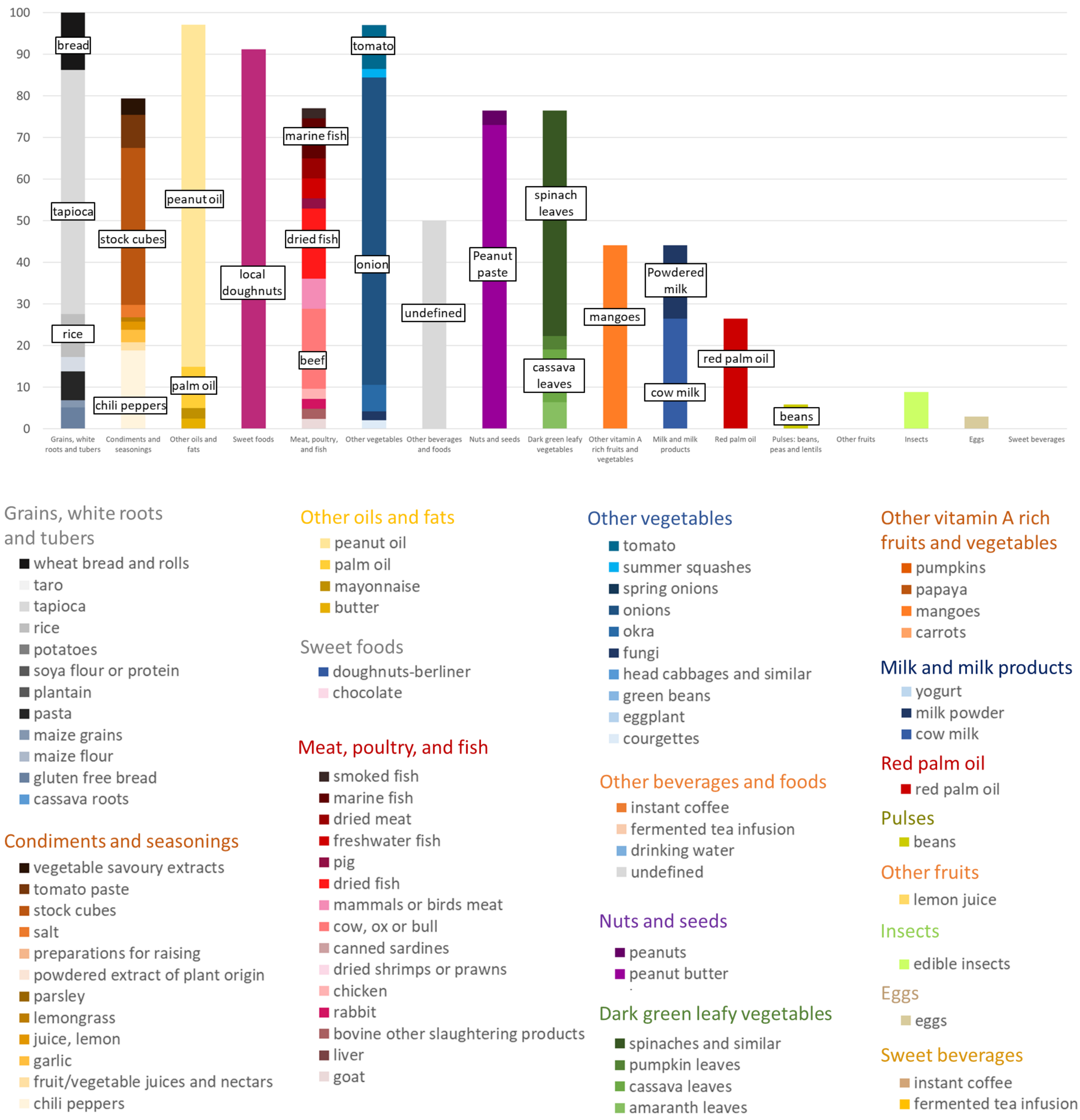

Y axis represents the % of women who consumed this food category at this specific time point out of all the women included in the study. Within each category, the relative importance of each food was calculated using the 24h-recall questionnaires.
